# An efficient access to the synthesis of novel 12-phenylbenzo[6,7]oxepino[3,4-*b*]quinolin-13(6*H*)-one derivatives

**DOI:** 10.3762/bjoc.8.213

**Published:** 2012-10-30

**Authors:** Wentao Gao, Guihai Lin, Yang Li, Xiyue Tao, Rui Liu, Lianjie Sun

**Affiliations:** 1 Institute of Superfine Chemicals, Bohai University, Jinzhou 121000, China

**Keywords:** Eaton’s reagent, Friedel–Crafts acylation, Friedländer reaction, one-pot, PPA, quinoline, tetracyclic-fused

## Abstract

An efficient access to the tetracyclic-fused quinoline systems, 12-phenylbenzo[6,7]oxepino[3,4-*b*]quinolin-13(6*H*)-one derivatives **4a**–**l**, is described, involving the intramolecular Friedel–Crafts acylation reaction of 2-(phenoxymethyl)-4-phenylquinoline-3-carboxylic acid derivatives **3a**–**l** aided by the treatment with PPA (polyphosphoric acid) or Eaton’s reagent. The required starting compound (**2**) was obtained by Friedländer reaction of 2-aminobenzophenone (**1**) with 4-chloroethylacetoacetate by using CAN (cerium ammonium nitrate, 10 mol %) as catalyst at room temperature. The substrates **3a**–**l** were prepared through one-pot reaction of ethyl 2-(chloromethyl)-4-phenylquinoline-3-carboxylate (**2**) and substituted phenols. Our developed strategy, involving a three-step route, offers easy access to tetracyclic-fused quinoline systems in short reaction times, and the products are obtained in moderate to good yields.

## Introduction

Polycyclic heterocycle-fused quinoline systems as important group compounds can be found in many biologically active natural products as well as in pharmacologically significant molecules, and have wide applications in medicinal chemistry [1–4]. It has been well-established that planar heterocycle-fused tri- or tetracyclic quinoline systems on privileged templates have significant biological properties, such as antitumoral [5–6], anti-inflammatory [7], antimalarial [8], antituberculosis [9], and antiplasmodial [10] activities. Accordingly, the synthesis of new families of such quinoline systems still attracts much interest from both medicinal and synthetic organic chemists [11–14]. Most reports in the literature contain a common five- or six-membered heterocycle fused to a quinoline ring, such as pyrazolo [15], pyrano [16], indolo [12,17], benzofuro [18], benzothienoquinolines [19], and synthetic analogues thereof. However, to the best of our knowledge, there are very few reports in which a medium-sized seven-membered benzoxepin ring is fused to a quinoline unit. In this context, Bera et al. [20] described a one-pot method for the synthesis of 6,7-dihydrobenzo[2,3]oxepino[4,5-*b*]quinolin-12-ols. However, the report is of episodic character and no efforts have been made to develop a general synthetic approach. Furthermore, the reported approach features a major restriction in the use of the expensive and unavailable 5-chloro-2,3-dihydrobenzo[*b*]oxepine-4-carbaldehyde as a reactant. Thus, a facile synthesis of such compounds by using inexpensive and readily available materials represents a challenging area for exploration.

On the other hand, the seven-membered benzoxepine nucleus can be found in many medicinally relevant natural products and synthetic compounds and represents one of the most profiled chemotypes in modern drug discovery, owing to several pronounced biological activities, such as antitumor and anti-inflammatory properties, attributed to the presence of the benzoxepine unit [21–24]. Last but not least, the benzoxepine nucleus has been of increasing relevance as a synthetic building block for the synthesis of manifold biologically and pharmaceutically important compounds [25–26]. As a consequence, the remarkable bioactivity surrounding the benzoxepine moiety has elicited a significant amount of interest as demonstrated by synthetic work already published [27–30].

In light of the above findings as well as the combination principles for drug design [31], we were intrigued to explore the incorporation of a quinoline ring fused together with a benzoxepine nucleus, which would be much more attractive and valuable for medicinal chemistry and drug discovery. In recent years, our research team has been interested in the development of efficient synthesis for the quinoline-based bioactive molecules [32–37]. Thus, in connection with our continuing interest in the synthesis of highly valuable quinoline compounds, we are actively involved in diversifying our work on the synthesis of hetero-fused quinoline systems that are of interest for medical research. Thus, the aim of the present work is to present the synthesis of novel compounds combining these two bioactive components in a molecular frame work as fused forms.

## Results and Discussion

**Scheme 1:**
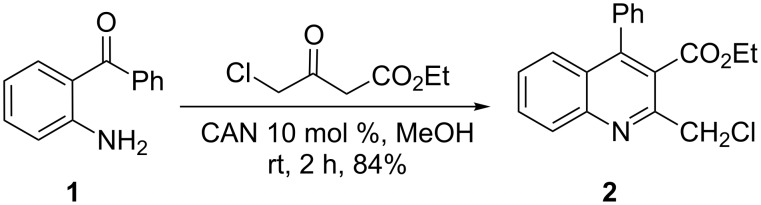
Synthesis of ethyl 2-(chloromethyl)-4-phenylquinoline-3-carboxylate (**2**).

The synthetic methodology developed in our laboratory for the synthesis of a new class of benzoxepino-fused quinoline compounds was achieved in a three-step procedure, commencing with the preparation of ethyl 2-(chloromethyl)-4-phenylquinoline-3-carboxylate (**2**) as shown in Scheme 1.

In fact, from the beginning, we were well aware that the preparation of ethyl 2-(chloromethyl)-4-phenylquinoline-3-carboxylate (**2**) is very interesting because such compounds are viewed as ideal starting materials for the flexible synthesis of a large range of quinoline derivatives [38–40]. Recently, Mizuno et al. [38] reported the preparation of ethyl 2-(chloromethyl)-4-(3,4-dimethoxyphenyl)-6,7-dimethoxy quinoline-3-carboxylate by the reaction of ethyl 4-chloroacetoacetate with 2-amino-3’,4,4’,5-tetramethoxybenzophenone hydrochloride in ethanol. However, in our case the reaction of 2-aminobenzophenone (**1**) with ethyl 4-chloroacetoacetate did not take place, and a gummy mass was obtained as product. In this context, Muscia et al. [39] described the synthesis of ethyl 6-chloro-2-(chloromethyl)-4-phenylquinoline-3-carboxylate by the Friedländer reaction employing microwave irradiation (MW) in the presence of a catalytic amount of hydrochloric acid. Subsequently, we reported a similar reaction under ultrasound irradiation conditions by using KHSO_4_ as catalyst [36]. Although the two methodologies are elegant and impressive, our attempts to follow both routes to synthesize **2** were frustrated by very low yields. In this regard, Bose et al. [40] reported the preparation of ethyl 6-chloro-2-(chloromethyl)-4-phenylquinoline-3-carboxylate by the treatment of 2-amino-5-chlorobenzophenone with 4-(chloroethyl)acetoacetate in MeOH by using 10 mol % CAN as catalyst at room temperature. To our delight, under similar reaction conditions, we were able to obtain **2** from 2-aminobenzophenone (**1**) and 4-(chloroethyl)acetoacetate in a good yield of 84% (Scheme 1). Moreover, the obtained product was very pure, and a chromatographic purification was unnecessary.

**Scheme 2:**
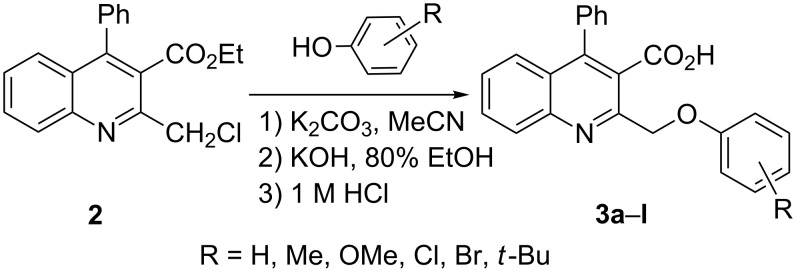
Synthesis of 2-(phenoxymethyl)-4-phenylquinoline-3-carboxylic acid derivatives **3a**–**l**.

Next, the resulting 2-(chloromethyl)quinoline **2** was subjected to the Williamson reaction with a variety of phenols with varying substituents in the presence of K_2_CO_3_ as base in MeCN under reflux as shown in Scheme 2.

In this reaction, we adopted MeCN as the solvent of choice simply because of its low boiling point, bringing much convenience to the workup procedure. Thus, upon the completion of the Williamson reaction as observed on TLC, MeCN was simply evaporated to dryness, 80% ethanolic potassium hydroxide solution (15 mL) was directly added to the residue, and the resulting reaction mixture was stirred under reflux. When the reactions were completed (usually within four hours) the quinoline-3-carboxylic acid ether compounds were obtained in high yields (80–93%) after simple recrystallization from ethanol. The structures assigned to these compounds are confirmed by spectral data and elemental analysis, which were fully consistent with the assigned molecular structure as depicted in Supporting Information File 1. The beauty of this reaction is that two chemical transformations, i.e., Williamson ether synthesis and subsequent ester hydrolysis take place in one-pot, thereby providing the acids in good yields of 66–93% with operational and experimental simplicity. Moreover, the presence of sterically hindered *tert*-butyl groups is not problematic although slightly lower yields were obtained when the aryl-ring was substituted in *o*-position by a *tert*-butyl group. The scope and generality of the newly synthesized compounds **3a**–**l** during the present investigation are listed in Table 1 together with yields and melting points.

**Table 1:** Yields and melting points of compounds **3a**–**l**.

Entry	Product	Yield (%)^a^	Mp (°C)

1	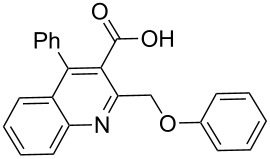 **3a**	83	191–192
2	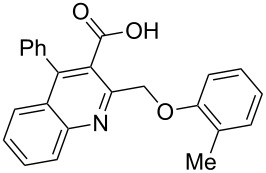 **3b**	91	209–210
3	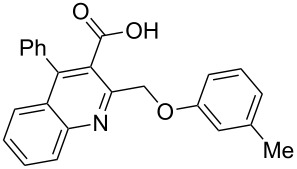 **3c**	88	212–213
4	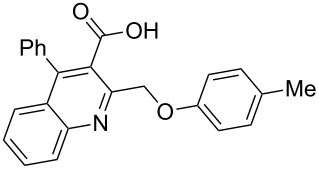 **3d**	93	207–208
5	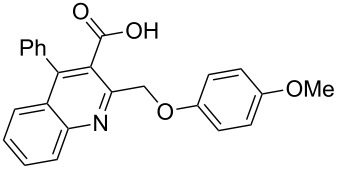 **3e**	86	197–198
6	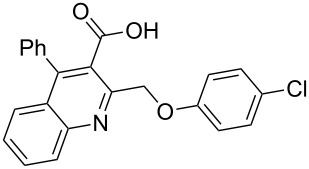 **3f**	83	167–168
7	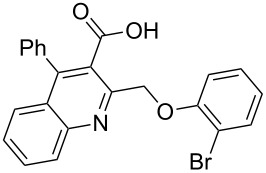 **3g**	88	165–166
8	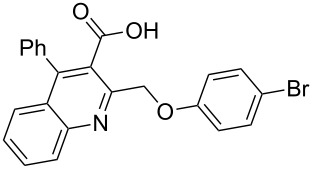 **3h**	82	196–197
9	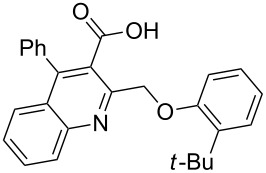 **3i**	71	192–194
10	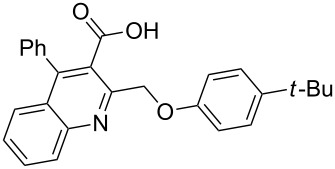 **3j**	85	188–190
11	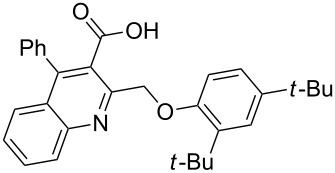 **3k**	66	230–232
12	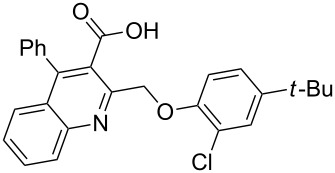 **3l**	82	239–241

^a^Isolated yield.

As shown in Table 1, high yields of **3** were achieved irrespective of the electronic nature or positions of the substituents, except for in the cases of **3i** and **3k** (Table 1, entries 9 and 11). The relatively lower yields of **3i** and **3k** may be ascribed to the sterically hindered nature of the bulky *tert*-butyl group at the *o*-position of aryl.

**Scheme 3:**
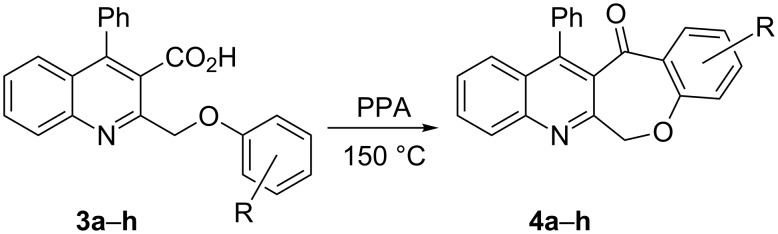
Synthesis of 12-phenylbenzo[6,7]oxepino[3,4-*b*]quinolin-13(6*H*)-ones **4a–h**.

Thus, the resulting substrates, quinoline-3-carboxylic acids **3a**–**l**, further served as active synthons for the intramolecular Friedel–Crafts acylation reaction to construct the desired tetracyclic benzoxepino-fused quinoline systems. Of the commonly available cyclization agents screened for the intramolecular Friedel–Crafts acylation reaction (e.g., AlCl_3_, H_2_SO_4_, *p*-TsOH, TiCl_4_, P_2_O_5_), the use of inexpensive and readily available polyphosphoric acid (PPA), requiring no additional solvent, was found to be very suitable for such a reaction in terms of good yield, short reaction time and simple workup [37,41]. Upon use of PPA as the cyclization agent, we found that the cyclization reaction of **3a**–**h** could be performed smoothly at 150 °C as shown in Scheme 3.

After the reaction was completed (monitored by TLC), the reaction mixture was poured into cold water to induce precipitation, followed by neutralization with NaHCO_3_ solution. Thus, the cyclized products **4a**–**h** were obtained in good yields, ranging from 69–85% after recrystallization from ethanol, and their identities were unequivocally ascertained from their satisfactory elemental and spectral data. The compounds **4a**–**h**, newly synthesized in the present investigation, are listed in Table 2.

**Table 2:** Structures and yields of the cyclized products **4a**–**h**.

Entry	Product	Time (h)	Yield (%)^a^	Mp (°C)

1	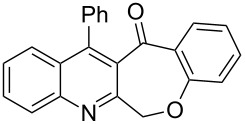 **4a**	6	77	197–198
2	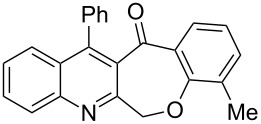 **4b**	5	80	215–216
3	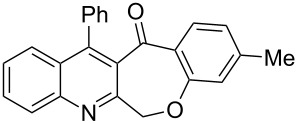 **4c**	5	83	199–200
4	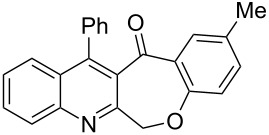 **4d**	5	82	195–197
5	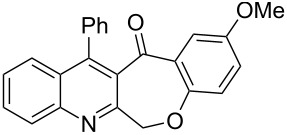 **4e**	5	85	197–199
6	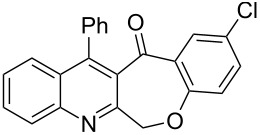 **4f**	7	74	175–177
7	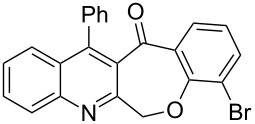 **4g**	7	69	171–172
8	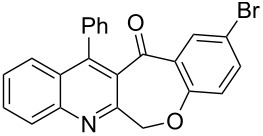 **4h**	7	72	179–180

^a^Isolated yield.

**Scheme 4:**
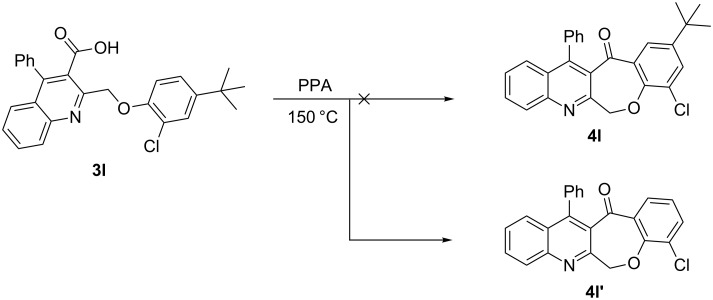
Cyclization and de-*tert*-butylation reaction of **3l** by using PPA.

As shown in Table 2, the cyclization reaction appears to be generally applicable, as most of the substrates **3** were consumed within 5–7 hours to give the corresponding cyclized products with reasonable yields. However, the method was limited due to difficulties in tolerating the *tert*-butyl groups. For example, when substrate **3l** was treated by PPA under the reaction conditions, the resulting product was assigned not to the expected **4l**, but characterized as the de-*tert*-butylation product **4l′** as shown in Scheme 4. The product was easily characterized from its ^1^H NMR spectrum, which showed no signals attributable to the carboxylic acid proton and *tert*-butyl protons of its precursor **3l**, along with the presence of a total count of twelve aromatic protons between 7.15–8.20 ppm, perfectly matched with their structures with additional support from its ^13^C NMR spectrum and other analytical data.

Similarly, in the present investigation the substrates **3i**–**k** also showed the same reaction patterns and the obtained product was identified as being the same structure as **4a**. Obviously, the problem with this is the loss of the *tert*-butyl group during the cyclization reaction course. In addition, slow and gradual warming to the reaction temperature was also tried, but this had no effect on the outcome of the reaction. Thus, according to our previous experience [33], coupled with the fact that the *tert*-butyl substituent could be easily removed from aromatic nuclei by Friedel–Crafts reaction at a high reaction temperature using a high-boiling-point solvent [42], we presumed the occurrence of debutylation reaction during the cyclization course probably due to the high reaction temperature.

Considering these results, an alternative route would be desirable. Recently, the Eaton’s reagent, a mixture of P_2_O_5_ and MeSO_3_H [43], has gained wide application as an advantageous medium for cyclization reactions [44], and we have also reported the serendipitous discovery of its excellent performance in these types of transformations [32–33,45]. Thus, we resorted to the use of our reliable approach to conduct the cyclization reaction. Under the same experimental conditions as in our previous methods, the ring closure of compounds **3i**–**l** was achieved and the corresponding cyclized products retaining the *tert*-butyl moiety were afforded in satisfactory yields of 59–70%. The reaction results are summarized in Table 3.

**Table 3:** Synthesis of *tert*-butyl-substituted compounds **4i**–**l**.

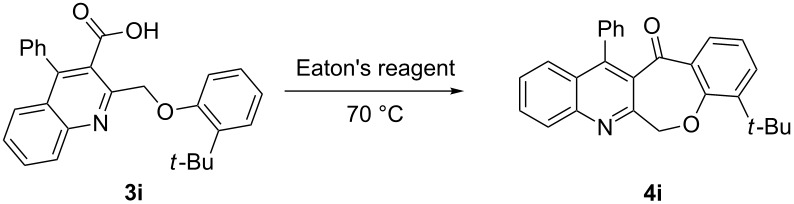				

Entry	Product	Time (h)	Yield (%)^a^	Mp (°C)

1	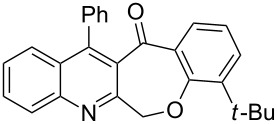 **4i**	6	68	193–195
2	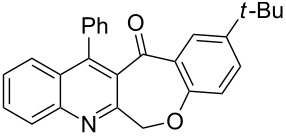 **4j**	5	70	210–212
3	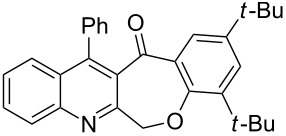 **4k**	5	62	166–168
4	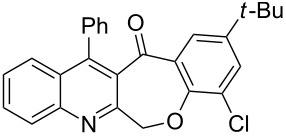 **4l**	5	59	209–210

^a^Isolated yield.

As shown in Table 3, by the treatment with Eaton’s reagent, the *tert*-butyl-substituted quinoline-3-carboxylic acids **3i**–**l** underwent the conversion smoothly and gave the corresponding cyclized products **4i**–**l** as expected. Compounds **4i**–**l** containing *tert*-butyl groups are interesting candidates for medicinal applications since it was reported that the introduction of *tert*-butyl groups into organic molecules could increase the lipophilicity of the molecule, which is very important for allowing passage through the extraordinarily thick and tight cell wall [46]. Altogether, the strategy using Eaton’s reagent instead of PPA showed a satisfactory conversion and gave us easy access to mono- and di-*tert*-butyl-substituted tetracyclic-fused quinoline systems. In addition, it is noteworthy that we also attempted additional experiments by conducting the cyclization reaction of **3a**–**l** under the given conditions (Eaton’s reagent, 70 °C). Although the cyclization reaction proceeded, the yields of the desired products were not as good as with PPA.

## Conclusion

In conclusion, we have described the synthesis of a series of structurally new 12-phenylbenzo[6,7]oxepino[3,4-*b*]quinolin-13(6*H*)-ones **4a**–**l**. The advantages of the current protocol include the ready availability of starting materials, ease of experimental operation, and satisfactory yields, which contribute to the usefulness of this method. These compounds belong to a new class of linearly fused tetracyclic heterocyclic quinoline systems, which could be potentially applied for the development of biologically and pharmaceutically important compounds. Access to such biologically intriguing structures should allow us to study their biological activities, and currently we are exploring this possibility.

## Experimental

All chemicals (AR graded) were commercially available and used without further purification. The melting points were determined by using a WRS-1B melting-points apparatus and were uncorrected. The IR spectra were obtained as KBr pellets in the range of 400–4000 cm^−1^ on a Shimadzu FTIR-8400S spectrophotometer (Shimadzu, Japan). ^1^H NMR and ^13^C NMR spectra were recorded on a Bruker AVANCE NMR spectrometer with CDCl_3_ or DMSO-*d*_6_ as the solvent. The reported chemical shifts (δ values) are given in parts per million (ppm) downfield from tetramethylsilane (TMS) as the internal standard. Mass spectra were determined on a MSD VL ESI1 spectrometer. Elemental analysis was recorded on an Elementar vario EL-III element analyzer.

**Procedure for the preparation of 2-(chloromethyl)-4-phenylquinoline-3-carboxylate (2)** [40]**.** To a stirred solution of 2-aminobenzophenone (**1**, 1.97 g, 10 mmol) and ethyl 4-chloroacetoacetate (1.65 g, 10 mmol) in methanol (15 mL), was added CAN (0.55 g, 1 mmol, 10 mol %). The resulting reaction mixture was stirred at room temperature for 2 h. After the reaction was completed (monitored by TLC), the mixture was washed with water (15 mL), extracted with ethyl acetate (30 × 2 mL), dried over Na_2_SO_4_, and concentrated under reduced pressure. The resulting residue was purified by silica gel column chromatography with petroleum ether/EtOAc (5:1) as eluent to afford 2.74 g of the product **2** as yellow crystals in 84% yield. Mp 103–105 °C (lit. [47] mp 109–111 °C).

**General procedure for the synthesis of 2-(phenoxymethyl)-4-phenylquinoline-3-carboxylic acid derivatives 3a–l.** A mixture of ethyl 2-(chloromethyl)-4-phenylquinoline-3-carboxylate (0.325 g, 1 mmol), substituted phenol (1 mmol) and K_2_CO_3_ (0.414 g, 3 mmol) was stirred in CH_3_CN (10 mL) under reflux. After completion of the reaction (monitored by TLC), CH_3_CN was evaporated to dryness. Then, a solution of KOH (2.8 g, 20 mmol) in 80% ethanol (15 mL) was added to the residue, and the mixture was heated under reflux for 4 h, cooled, and acidified with 1 M hydrochloric acid solution. The resulting crude product was recrystallized from ethanol to afford **3a**–**l**. The yields and melting points of all compounds are summarized in Table 1 and the spectral and analytical data are given in Supporting Information File 1.

**General procedure for the synthesis of 12-phenylbenzo[6,7]oxepino[3,4-*b*]quinolin-13(6*H*)-one derivatives 4a–h.** The precursors 2-(phenoxymethyl)-4-phenylquinoline-3-carboxylic acid derivatives (**3a**–**h**, 0.5 mmol) and PPA (10 g) were added to a round flask (25 mL) and stirred at 150 °C for 5–7 h. The conversion was monitored by TLC. After the reaction was completed, the reaction mixture was poured slowly into cold water under stirring to induce precipitation, followed by neutralization with NaHCO_3_ solution. The obtained crude products were recrystallized from ethanol to afford products **4a**–**h**. The yields are summarized in Table 2 and the spectra and analytical data are given in Supporting Information File 1.

**General procedure for the synthesis of *tert*-butyl-substituted 12-phenylbenzo[6,7]oxepino[3,4-*b*]quinolin-13(6*H*)-one derivatives 4i–l.** The *tert*-butyl-substituted precursors 2-(phenoxymethyl)-4-phenylquinoline-3-carboxylic acid derivatives (**3i**–**l**, 0.5 mmol) and Eaton’s reagent (5 mL) were added to a round flask (10 mL) and stirred at 70 °C for 5–6 h. The conversion was monitored by TLC. After the reaction was completed, the reaction mixture was poured slowly into cold water under stirring to induce precipitation, followed by neutralization with NaHCO_3_ solution. The crude products were obtained after filtration and washing with water. The pure products **4i**–**l** were obtained by recrystallization from ethanol. The yields are summarized in Table 3 and the spectra and analytical data are given in Supporting Information File 1.

## Supporting Information

File 1 Characterization data of the title compounds and NMR and HRMS spectra.
