# Supplementary material for: An efficient access to the synthesis of novel 12-phenylbenzo[6,7]oxepino[3,4-b]quinolin-13(6H)-one derivatives
Source: Beilstein J Org Chem. 2012 Oct 30;8:1849–57. doi: 10.3762/bjoc.8.213 (PMC3511022; doi:10.3762/bjoc.8.213)
Supplement: File 1 — Characterization data of the title compounds and NMR and HRMS spectra. [file Beilstein_J_Org_Chem-08-1849-s001.pdf]

Supporting Information

for

An efficient access to the synthesis of novel 12-phenylbenzo[6,7]oxepino[3,4-*b*]quinolin-13(6*H*)-one derivatives

Wentao Gao*, Guihai Lin, Yang Li, Xiyue Tao, Rui Liu and Lianjie Sun

Address: Institute of Superfine Chemicals, Bohai University, Jinzhou 121000, China

Email: Wentao Gao* - bhuzh@163.com

* Corresponding author

Characterization data of the title compounds and NMR and HRMS spectra

Characterization data for products 3a–l

2-(Phenoxymethyl)-4-phenylquinoline-3-carboxylic acid (3a). Yellow solid, mp 191–192 °C; IR (KBr) v/cm^{-1}: 3064, 2922, 1942, 1723, 1599, 1488, 1396, 1239, 1174, 1073, 1046, 929, 883, 727; ^{1}H NMR (600 MHz, DMSO-d_6) δ (ppm): 13.16 (s, 1H, COOH), 8.14 (d, J = 8.4 Hz, 1H, ArH), 7.86 (t, J = 7.7 Hz, 1H, ArH), 7.63 (t, J = 7.6 Hz, 1H, ArH), 7.50–7.57 (m, 4H, ArH), 7.38–7.39 (m, 2H, ArH), 7.30 (t, J = 7.9 Hz, 2H, ArH), 7.03 (d, J = 8.0 Hz, 2H, ArH), 6.96 (t, J = 7.3 Hz, 1H, ArH), 5.43 (s, 2H, CH_2); ^{13}C NMR (150 MHz, DMSO-d_6) δ (ppm): 70.82, 114.84, 121.19, 125.86, 126.25, 127.66, 128.00, 128.38, 128.62, 129.22, 129.32, 129.57, 130.71, 135.13, 145.94, 146.39, 152.86, 158.31, 168.27; MS (ESI, *m/z*): 356.1 $[M + H]^+$; Anal. calcd for $C_{23}H_{17}NO_3$: C, 77.73; H, 4.82; N, 3.94; found: C, 77.60; H, 4.98; N, 3.80.

2-[(2-Methylphenoxy)methyl]-4-phenylquinoline-3-carboxylic acid (3b). White crystals, mp 209–210 °C; IR (KBr) v/cm^{-1}: 3029, 2739, 1707, 1570, 1494, 1442, 1242, 1178, 1069, 1029, 925, 885, 728; ^{1}H NMR (600 MHz, DMSO-d_6) δ (ppm): 13.13 (s, 1H, COOH), 8.15 (d, J = 8.4 Hz, 1H, ArH), 7.87 (t, J = 7.6 Hz, 1H, ArH), 7.64 (t, J = 7.6 Hz, 1H, ArH), 7.51–7.56 (m, 4H, ArH), 7.38–7.40 (m, 2H, ArH), 7.012 (dt, J = 13.1, 7.9 Hz, 3H, ArH), 6.84 (t, J = 7.3 Hz, 1H, ArH), 5.44 (s, 2H, CH_2), 2.12 (s, 3H, CH_3); ^{13}C NMR (150 MHz, DMSO-d_6) δ (ppm): 16.03, 70.81, 111.23, 120.65, 125.91, 126.25, 126.28, 126.93, 128.01, 128.36, 128.58, 129.23, 129.30, 130.56, 130.72, 135.20, 146.09, 146.37, 153.08, 156.36, 168.27; MS (ESI, *m/z*): 370.1 $[M + H]^+$; Anal. calcd for $C_{24}H_{19}NO_3$: C, 78.03; H, 5.18; N, 3.79; found: C, 78.25; H, 5.01; N, 3.64.

2-[(3-Methylphenoxy)methyl]-4-phenylquinoline-3-carboxylic acid (3c). White crystals, mp 212–213 °C; IR (KBr) ν/cm^{-1}: 3033, 2916, 1712, 1571, 1488, 1443, 1230, 1171, 1071, 1047, 921, 878, 730; ^{1}H NMR (600 MHz, DMSO-d_6) δ (ppm): 13.14 (s, 1H, COOH), 8.14 (d, *J* = 8.4 Hz, 1H, ArH), 7.86 (ddd, *J* = 8.3, 6.9, 1.3 Hz, 1H, ArH), 7.62 (ddd, *J* = 8.2, 6.9, 1.1 Hz, 1H, ArH), 7.50–7.57 (m, 4H, ArH), 7.38–7.40 (m, 2H, ArH), 7.17 (t, *J* = 7.8 Hz, 1H, ArH), 6.86 (s, 1H, ArH), 6.83 (dd, *J* = 8.2, 2.3 Hz, 1H, ArH), 6.77 (d, *J* = 7.5 Hz, 1H, ArH), 5.40 (s, 2H, CH_2), 2.27 (s, 3H, CH_3); ^{13}C NMR (150 MHz, DMSO-d_6) δ (ppm): 21.20, 70.76, 111.71, 115.56, 121.94, 125.86, 126.24, 127.70, 127.98, 128.38, 128.61, 129.22, 129.32, 130.70, 135.15, 139.04, 145.91, 146.39, 152.89, 158.35, 168.24; MS (ESI, *m/z*): 370.1 [M + H]$^+$; Anal. calcd for $C_{24}H_{19}NO_3$: C, 78.03; H, 5.18; N, 3.79; found: C, 78.22; H, 5.05; N, 3.62.

2-[(4-Methylphenoxy)methyl]-4-phenylquinoline-3-carboxylic acid (3d). White crystals, mp 207–208 °C; IR (KBr) ν/cm^{-1}: 3059, 2919, 1706, 1572, 1488, 1450, 1380, 1245, 1178, 1069, 1039, 929, 874, 729; ^{1}H NMR (600 MHz, DMSO-d_6) δ (ppm): 13.14 (s, 1H, COOH), 8.13 (d, *J* = 8.2 Hz, 1H, ArH), 7.84–7.87 (m, 1H, ArH), 7.60–7.63 (m, 1H, ArH), 7.49–7.57 (m, 4H, ArH), 7.38 (dd, *J* = 7.7, 1.5 Hz, 2H, ArH), 7.08 (d, *J* = 8.4 Hz , 2H, ArH), 6.91 (d, *J* = 8.6 Hz, 2H, ArH), 5.39 (s, 2H, CH_2), 2.22 (s, 3H, CH_3); ^{13}C NMR (150 MHz, DMSO-d_6) δ (ppm): 20.17, 70.93, 114.74, 125.85, 126.24, 127.97, 128.39, 128.62, 129.22, 129.32, 129.88, 130.69, 135.14, 145.89, 146.38, 153.02, 156.24, 168.26; MS (ESI, *m/z*): 370.1 [M + H]$^+$; Anal. calcd for $C_{24}H_{19}NO_3$: C, 78.03; H, 5.18; N, 3.79; found: C, 78.20; H, 5.01; N, 3.60.

2-[(4-Methoxyphenoxy)methyl]-4-phenylquinoline-3-carboxylic acid (3e). White crystals, mp 197–198 °C; IR (KBr) ν/cm^{-1}: 3067, 2840, 1718, 1584, 1508, 1444, 1226, 1186, 1096, 1032, 928, 899, 734; ^{1}H NMR (600 MHz, DMSO-d_6) δ (ppm): 13.18 (s, 1H, COOH), 8.14 (d, J = 8.3 Hz, 1H, ArH), 7.86 (t, J = 7.5 Hz, 1H, ArH), 7.62 (t, J = 7.5 Hz, 1H, ArH), 7.46–7.59 (m, 4H, ArH), 7.39 (d, J = 6.4 Hz, 2H, ArH), 6.92–7.20 (m, 2H, ArH), 6.74–6.92 (m, 2H, ArH), 5.38 (s, 2H, CH_2), 3.69 (s, 3H, OCH_3); ^{13}C NMR (150 MHz, DMSO-d_6) δ (ppm): 55.46, 71.25, 114.68, 115.94, 125.94, 126.32, 127.74, 128.12, 128.42, 128.71, 128.83, 129.32, 130.93, 135.07, 145.93, 146.36, 152.31, 153.05, 153.94, 168.14; MS (ESI, m/z): 386.1 [M + H]$^+$; Anal. calcd for $C_{24}H_{19}NO_4$: C, 74.79; H, 4.97; N, 3.63; found: C, 74.58; H, 5.17; N, 3.45.

2-[(4-Chlorophenoxy)methyl]-4-phenylquinoline-3-carboxylic acid (3f). White crystals, mp 167–168 °C; IR (KBr) ν/cm^{-1}: 3058, 2871, 1716, 1575, 1487, 1389, 1244, 1167, 1071, 1040, 929, 879, 726; ^{1}H NMR (600 MHz, DMSO-d_6) δ (ppm): 13.20 (s, 1H, COOH), 8.13 (d, J = 8.4 Hz, 1H, ArH), 7.86 (t, J = 7.6 Hz, 1H, ArH), 7.63 (t, J = 7.6 Hz, 1H, ArH), 7.53–7.60 (m, 4H, ArH), 7.46 (d, J = 8.8 Hz, 2H, ArH), 7.38 (d, J = 6.2 Hz, 2H, ArH), 7.00 (d, J = 8.9 Hz, 2H, ArH), 5.43 (s, 2H, CH_2); ^{13}C NMR (150 MHz, DMSO-d_6) δ (ppm): 71.10, 112.70, 117.17, 125.86, 126.24, 127.59, 128.05, 128.37, 128.63, 129.22, 129.30, 130.74, 132.23, 135.08, 145.99, 146.37, 152.45, 157.58, 168.23; MS (ESI, m/z): 390.1 [M + H]$^+$; Anal. calcd for $C_{23}H_{16}ClNO_3$: C, 70.86; H, 4.14; N, 3.59; found: C, 70.65; H, 4.32; N, 3.36.

2-[(2-Bromophenoxy)methyl]-4-phenylquinoline-3-carboxylic acid (3g). Yellow solid, mp 165–166 °C; IR (KBr) ν/cm^{-1}: 3063, 2919, 1706, 1570, 1477, 1389, 1235, 1178, 1070, 1032, 929, 886, 725; ^{1}H NMR (600 MHz, DMSO-d_6) δ (ppm): 13.19 (s, 1H, COOH), 8.14 (d, J = 7.6 Hz, 1H, ArH), 7.87 (t, J = 6.7 Hz, 1H, ArH), 7.63 (t, J = 6.7 Hz, 1H, ArH), 7.54–7.58 (m, 5H, ArH), 7.36–7.41 (m, 2H, ArH), 7.26–7.34 (m, 2H, ArH), 6.92 (t, J = 6.7 Hz, 1H, ArH), 5.52 (s, 2H, CH_2); ^{13}C NMR (150 MHz, DMSO-d_6) δ (ppm): 71.59, 111.28, 114.14, 114.84, 122.60, 125.94, 126.30, 127.51, 128.15, 128.39, 128.62, 129.01, 129.30, 129.57, 130.82, 133.23, 135.24, 146.28, 146.47, 152.16, 154.70, 168.12; MS (ESI, *m/z*): 434.0 [M + H]$^+$; Anal. calcd for $C_{23}H_{16}BrNO_3$: C, 63.61; H, 3.71; N, 3.23; found: C, 63.42; H, 3.88; N, 3.03.

2-[(4-Bromophenoxy)methyl]-4-phenylquinoline-3-carboxylic acid (3h). White solid, mp 196–197 °C; IR (KBr) ν/cm^{-1}: 3057, 2870, 1716, 1573, 1486, 1389, 1243, 1167, 1070, 1039, 929, 878, 726; ^{1}H NMR (600 MHz, DMSO-d_6) δ (ppm): 13.21 (s, 1H, COOH), 8.12 (d, J = 8.4 Hz, 1H, ArH), 7.85 (t, J = 7.5 Hz, 1H, ArH), 7.61 (t, J = 7.6 Hz, 1H, ArH), 7.49–7.55 (m, 4H, ArH), 7.45 (d, J = 8.9 Hz, 2H, ArH), 7.37 (d, J = 7.5 Hz, 2H, ArH), 6.99 (d, J = 8.9 Hz, 2H, ArH), 5.42 (s, 2H, CH_2); ^{13}C NMR (150 MHz, DMSO-d_6) δ (ppm): 71.08, 112.70, 117.18, 125.87, 126.24, 128.04, 128.38, 128.62, 129.22, 129.32, 130.71, 132.23, 135.11, 146.36, 152.46, 157.59, 168.26; MS (ESI, *m/z*): 434.0 [M + H]$^+$; Anal. calcd for $C_{23}H_{16}BrNO_3$: C, 63.61; H, 3.71; N, 3.23; found: C, 63.40; H, 3.90; N, 3.01.

2-[(2-*tert*-Butylphenoxy)methyl]-4-phenylquinoline-3-carboxylic acid (3i). Yellow solid, mp 192–194 °C; IR (KBr) ν/cm^{-1}: 3032, 2959, 1727, 1560, 1489, 1441, 1231, 1179, 1094, 1033, 926, 888, 725; ^{1}H NMR (600 MHz, DMSO-d_6) δ (ppm): 13.26 (s, 1H, COOH), 8.13 (d, J = 8.4 Hz, 1H, ArH), 7.86 (t, J = 7.6 Hz, 1H, ArH), 7.62 (t, J = 7.6 Hz, 1H, ArH), 7.52–7.56 (m, 4H, ArH), 7.39 (d, J = 6.7 Hz, 2H, ArH), 7.22 (d, J = 7.7 Hz, 1H, ArH), 7.16 (d, J = 3.7 Hz, 2H, ArH), 6.89 (m, 1H, ArH), 5.44 (s, 2H, CH_2), 1.29 (s, 9H, *tert*-butyl); ^{13}C NMR (150 MHz, DMSO-d_6) δ (ppm): 29.93, 34.52, 70.88, 113.27, 120.88, 125.75, 126.27, 126.42, 127.24, 128.02, 128.41, 128.62, 129.34, 130.75, 135.28, 137.86, 146.05, 146.70, 152.73, 157.58, 168.28; MS (ESI, *m/z*): 412.2 [M + H]$^+$; Anal. calcd for $C_{27}H_{25}NO_3$: C, 78.81; H, 6.12; N, 3.40; found: C, 78.59; H, 6.31; N, 3.25.

2-[(4-*tert*-Butylphenoxy)methyl]-4-phenylquinoline-3-carboxylic acid (3j). Yellow crystals, mp 188–190 °C; IR (KBr) ν/cm^{-1}: 3076, 2953, 1707, 1569, 1509, 1390, 1241, 1181, 1068, 1037, 932, 889, 728; ^{1}H NMR (600 MHz, DMSO-d_6) δ (ppm): 13.11 (s, 1H, COOH), 8.13 (d, J = 8.4 Hz, 1H, ArH), 7.85 (t, J = 7.6 Hz, 1H, ArH), 7.61 (t, J = 7.6 Hz, 1H, ArH), 7.48–7.55 (m, 4H, ArH), 7.37–7.38 (m, 2H, ArH), 7.28 (d, J = 8.7 Hz , 2H, ArH), 6.93 (d, J = 8.7 Hz, 2H, ArH), 5.39 (s, 2H, CH_2), 1.23 (s, 9H, *tert*-butyl); ^{13}C NMR (150 MHz, DMSO-d_6) δ (ppm): 31.29, 33.78, 70.75, 114.18, 125.76, 126.09, 126.14, 127.88, 128.28, 128.51, 129.12, 129.22, 130.59, 135.03, 143.26, 146.26, 152.98, 155.97, 168.14; MS (ESI, *m/z*):412.2 [M + H]$^+$; Anal. calcd for $C_{27}H_{25}NO_3$: C, 78.81; H, 6.12; N, 3.40; found: C, 78.62; H, 6.33; N, 3.28.

2-[(2,4-Di-*tert*-butylphenoxy)methyl]-4-phenylquinoline-3-carboxylic acid (3k). White crystals, mp 230–232 °C; IR (KBr) ν/cm^{-1}: 3075, 2957, 1707, 1570, 1497, 1456, 1232, 1179, 1070, 1034, 930, 887, 730; ^{1}H NMR (600 MHz, DMSO-d_6) δ (ppm): 13.23 (s, 1H, COOH), 8.14 (d, *J* = 8.4 Hz, 1H, ArH), 7.87 (t, *J* = 7.6 Hz, 1H, ArH), 7.64 (t, *J* = 7.7 Hz, 1H, ArH), 7.53–7.57 (m, 4H, ArH), 7.40 (d, *J* = 6.7 Hz, 2H, ArH), 7.24 (s, 1H, ArH), 7.16 (dd, *J* = 8.5, 1.9 Hz, 1H, ArH), 7.08 (d, *J* = 8.5 Hz, 1H, ArH), 5.42 (s, 2H, CH_2), 1.31 (s, 9H, *tert*-butyl), 1.25 (s, 9H, *tert*-butyl); ^{13}C NMR (150 MHz, DMSO-d_6) δ (ppm): 30.01, 31.47, 34.01, 34.66, 71.02, 112.86, 123.13, 123.57, 125.76, 126.25, 127.56, 127.98, 128.39, 128.59, 129.32, 130.72, 135.28, 137.00, 142.60, 146.04, 146.66, 153.00, 155.31, 168.22; MS (ESI, *m/z*): 468.3 [M + H]$^+$; Anal. calcd for $C_{31}H_{33}NO_3$: C, 79.63; H, 7.11; N, 3.00; found: C, 79.47; H, 7.29; N, 3.21.

2-[(4-(*tert*-Butyl)-2-chlorophenoxy)methyl]-4-phenylquinoline-3-carboxylic acid (3l). Yellow crystals. mp 188–189 °C; IR (KBr) ν/cm^{-1}: 3075, 2961, 1711, 1568, 1490, 1454, 1247, 1180, 1069, 1032, 931, 883, 727; ^{1}H NMR (600 MHz, DMSO-d$_6$) δ (ppm): ^{1}H NMR (600 MHz, DMSO-d_6) δ (ppm): 13.14 (s, 1H, COOH), 8.13 (d, *J* = 8.4 Hz, 1H, ArH), 7.86 (t, *J* = 7.6 Hz, 1H, ArH), 7.63 (t, *J* = 7.6 Hz, 1H, ArH), 7.50-7.55 (m, 4H, ArH), 7.37 (d, *J* = 8.5 Hz, 3H, ArH), 7.27 (d, *J* = 8.7 Hz , 1H, ArH), 7.21 (d, *J* = 8.7 Hz, 1H, ArH), 5.47 (s, 2H, CH_2), 1.23 (s, 9H, *tert*-butyl); ^{13}C NMR (150 MHz, DMSO-d_6) δ (ppm): 31.33, 34.24, 71.76, 113.98, 121.49, 125.11, 126.15, 126.49, 127.25, 127.75, 128.33, 128.59, 128.82, 129.49, 131.00, 135.42, 144.99, 146.42, 146.64, 151.71, 152.58, 168.32; MS (ESI, *m/z*): 446.1 [M + H]$^+$; Anal. calcd for $C_{27}H_{24}ClNO_3$: C, 72.72; H, 5.42; N, 3.14; found: C, 72.51; H, 5.61; N, 2.97.

Characterization data for products 4a–l

12-Phenylbenzo[6,7]oxepino[3,4-*b*]quinolin-13(6*H*)-one (4a). White solid, mp 197–198 °C; IR (KBr) ν/cm^{-1}: 3064, 1960, 1560, 1474, 1401, 1298, 1145, 1028, 969, 760, 703; ^{1}H NMR (600 MHz, DMSO-d_6) δ (ppm): 8.20 (d, *J* = 8.4 Hz, 1H, ArH), 7.92 (t, *J* = 7.2 Hz, 1H, ArH), 7.65 (t, *J* = 7.4 Hz, 1H, ArH), 7.55–7.61 (m, 2H, ArH), 7.50–7.54 (m, 4H, ArH), 7.21 (dd, *J* = 6.4, 2.8 Hz, 2H, ArH), 7.15 (t, *J* = 7.7 Hz, 2H, ArH), 5.53 (s, 2H, CH_2); ^{13}C NMR (150 MHz, DMSO-d_6) δ (ppm): 75.33, 120.01, 122.26, 126.59, 126.68, 127.02, 128.23, 128.36, 128.41, 129.20, 129.38, 130.20, 131.54, 132.82, 135.16, 135.50, 147.80, 148.35, 152.74, 159.34, 192.03; MS (ESI, *m/z*): 338.1 [M + H]$^+$; Anal. calcd for $C_{23}H_{15}NO_2$: C, 81.88; H, 4.48; N, 4.15; found: C, 81.67; H, 4.65; N, 4.00.

4-Methyl-12-phenylbenzo[6,7]oxepino[3,4-*b*]quinolin-13(6*H*)-one (4b). White crystals, mp 215–216 °C; IR (KBr) ν/cm^{-1}: 3059, 1707, 1594, 1468, 1378, 1256, 1135, 1035, 906, 763, 700; ^{1}H NMR (600 MHz, $CDCl_3$) δ (ppm): ^{1}H NMR (600 MHz, $CDCl_3$) δ (ppm): 8.12 (d, *J* = 8.4 Hz, 1H, ArH), 7.71 (dd, *J* = 8.2, 7.0 Hz, 1H, ArH), 7.59 (d, *J* = 8.5 Hz, 1H, ArH), 7.46–7.51 (m, 1H, ArH), 7.36–7.45 (m, 4H, ArH), 7.15–7.19 (m, 2H, ArH), 6.77–6.81 (m, 2H, ArH), 5.40 (s, 2H, CH_2), 2.28 (s, 3H, CH_3); ^{13}C NMR (150 MHz, $CDCl_3$) δ (ppm): 16.51, 75.52, 121.45, 126.90, 127.41, 127.54, 128.27, 128.38, 128.42, 129.04, 129.34, 129.58, 130.91, 133.48, 135.49, 135.96, 148.40, 148.70, 152.64, 157.96, 193.64; MS (ESI, *m/z*): 352.1 [M + H]$^+$; Anal. calcd for $C_{24}H_{17}NO_2$: C, 82.03; H, 4.88; N, 3.99; found: C, 82.31; H, 4.69; N, 3.78.

3-Methyl-12-phenylbenzo[6,7]oxepino[3,4-*b*]quinolin-13(6*H*)-one (4c). White crystals, mp 199–200 °C; IR (KBr) ν/cm^{-1}: 3060, 1667, 1565, 1488, 1377, 1297, 1164, 1042, 969, 770, 705; ^{1}H NMR (600 MHz, $CDCl_3$) δ (ppm): 8.19 (d, *J* = 8.4 Hz, 1H, ArH), 7.78 (t, *J* = 7.6 Hz, 1H, ArH), 7.66 (d, *J* = 8.3 Hz, 1H, ArH), 7.56 (d, *J* = 8.3 Hz, 1H, ArH), 7.47–7.52 (m, 4H, ArH), 7.23–7.25 (m, 2H, ArH), 6.88 (d, *J* = 7.2 Hz, 2H, ArH), 5.48 (s, 2H, CH_2), 2.36 (s, 3H, CH_3); ^{13}C NMR (150 MHz, $CDCl_3$) δ (ppm): 21.47, 75.69, 119.96, 123.35, 124.72, 127.41, 127.50, 128.23, 128.30, 129.27, 129.57, 130.59, 130.90, 133.35, 135.68, 146.39, 148.33, 148.91, 152.68, 159.83, 192.64; MS (ESI, *m/z*): 352.1 $[M + H]^+$; Anal. calcd for $C_{24}H_{17}NO_2$: C, 82.03; H, 4.88; N, 3.99; found: C, 82.21; H, 4.66; N, 4.12.

2-Methyl-12-phenylbenzo[6,7]oxepino[3,4-*b*]quinolin-13(6*H*)-one (4d). White crystals, mp 195–197 °C; IR (KBr) ν/cm^{-1}: 3050, 1707, 1566, 1489, 1402, 1256, 1126, 1027, 981, 763, 697; ^{1}H NMR (600 MHz, $CDCl_3$) δ (ppm): 8.19 (d, *J* = 8.4 Hz, 1H, ArH), 7.78 (dd, *J* = 11.2, 4.0 Hz, 1H, ArH), 7.65 (d, *J* = 8.3 Hz, 1H, ArH), 7.48–7.52 (m, 4H, ArH), 7.44 (s, 1H, ArH), 7.29 (dd, *J* = 8.4, 2.1 Hz, 1H, ArH), 7.25 (dd, *J* = 6.5, 2.9 Hz, 2H, ArH), 6.97 (d, *J* = 8.4 Hz, 1H, ArH), 5.46 (s, 2H, CH_2), 2.30 (s, 3H, CH_3); ^{13}C NMR (150 MHz, $CDCl_3$) δ (ppm): 20.30, 75.77, 119.89, 126.56, 127.43, 127.50, 128.23, 128.31, 129.31, 129.56, 130.17, 130.92, 131.45, 133.27, 135.62, 136.08, 148.34, 148.96, 152.72, 157.95, 193.23; MS (ESI, *m/z*): 352.1 $[M + H]^+$; Anal. calcd for $C_{24}H_{17}NO_2$: C, 82.03; H, 4.88; N, 3.99; found: C, 82.18; H, 4.70; N, 4.16.

2-Methoxy-12-phenylbenzo[6,7]oxepino[3,4-*b*]quinolin-13(6*H*)-one (4e). White crystals, mp 197–199 °C; IR (KBr) ν/cm^{-1}: 3066, 1706, 1559, 1465, 1397, 1306, 1177, 1047, 910, 763, 684; ^{1}H NMR (600 MHz, $CDCl_3$) δ (ppm): 8.22 (d, *J* = 8.4 Hz, 1H, ArH), 7.82 (dd, *J* = 8.2, 7.1 Hz, 1H, ArH), 7.69 (d, *J* = 8.5 Hz, 1H, ArH), 7.56–7.48 (m, 4H, ArH), 7.30–7.25 (m, 2H, ArH), 7.11 (dd, *J* = 6.7, 4.6 Hz, 2H, ArH), 7.04 (d, *J* = 8.2 Hz, 1H, ArH), 5.48 (s, 2H, CH_2), 3.78 (s, 3H, OCH_3); ^{13}C NMR (150 MHz, $CDCl_3$) δ (ppm): 55.87, 75.78, 111.49, 121.43, 123.74, 126.62, 127.45, 127.63, 128.26, 128.41, 129.27, 129.29, 131.17, 132.88, 135.57, 152.67, 154.38, 154.53, 192.61; MS (ESI, *m/z*): 368.1 [M + H]$^+$; Anal. calcd for $C_{24}H_{17}NO_3$: C, 78.46; H, 4.66; N, 3.81; found: C, 78.28; H, 4.75; N, 3.67.

2-Chloro-12-phenylbenzo[6,7]oxepino[3,4-*b*]quinolin-13(6*H*)-one (4f). Yellow solid, mp 175–177 °C; IR (KBr) ν/cm^{-1}: 3066, 1706, 1559, 1488, 1397, 1230, 1177, 1047, 939, 763, 718; ^{1}H NMR (600 MHz, $CDCl_3$) δ (ppm): 8.51 (d, *J* = 8.4 Hz, 1H, ArH), 8.20 (d, *J* = 8.4 Hz, 1H, ArH), 8.15 (d, *J* = 7.6 Hz, 1H, ArH), 7.85–7.88 (m, 1H, ArH), 7.76 (d, *J* = 7.2 Hz, 1H, ArH), 7.70 (t, *J* = 7.2 Hz, 1H, ArH), 7.63-7.65 (m, 1H, ArH), 7.51 (t, *J* = 7.2 Hz, 1H, ArH), 7.37–7.39 (m, 2H, ArH), 6.99-7.01 (m, 2H, ArH), 5.70 (s, 2H, CH_2); ^{13}C NMR (150 MHz, $CDCl_3$) δ (ppm): 68.15, 113.30, 117.06, 122.90, 123.53, 124.57, 124.74, 124.82, 128.41, 131.15, 131.28, 132.19, 132.55, 133.92, 134.73, 142.10, 151.78, 152.59, 153.09, 158.13, 193.03; MS (ESI, *m/z*): 372.1 [M + H]$^+$; Anal. calcd for $C_{23}H_{14}ClNO_2$: C, 74.30; H, 3.80; N, 3.77; found: C, 74.12; H, 3.99; N, 3.64.

4-Bromo-12-phenylbenzo[6,7]oxepino[3,4-*b*]quinolin-13(6*H*)-one (4g). Yellow solid, mp 171–172 °C; IR (KBr) ν/cm^{-1}: 3065, 1707, 1570, 1480, 1397, 1238, 1191, 1051, 906, 766, 711; ^{1}H NMR (600 MHz, $CDCl_3$) δ (ppm): 8.52 (d, *J* = 8.4 Hz, 1H, ArH), 8.21 (d, *J* = 8.6 Hz, 1H, ArH), 8.15 (d, *J* = 7.6 Hz, 1H, ArH), 7.86 (t, *J* = 7.2 Hz, 1H, ArH), 7.77 (d, *J* = 7.2 Hz, 1H, ArH), 7.71 (t, *J* = 7.6 Hz, 1H, ArH), 7.64 (t, *J* = 7.1 Hz, 1H, ArH), 7.54 (dd, *J* = 7.9, 1.5 Hz, 1H, ArH), 7.49–7.52 (m, 2H, ArH), 7.25–7.28 (m, 1H, ArH), 6.85 (t, *J* = 7.0 Hz, 1H, ArH), 5.78 (s, 2H, CH_2); ^{13}C NMR (150 MHz, DMSO-d_6) δ (ppm): 68.87, 111.19, 114.46, 122.40, 122.79, 122.86, 124.19, 125.64, 125.71, 129.05, 130.36, 131.81, 133.08, 133.17, 133.23, 134.73, 135.52, 141.21, 150.95, 152.12, 152.42, 155.16, 192.37; MS (ESI, *m/z*): 416.0 [M + H]$^+$; Anal. calcd for $C_{23}H_{14}BrNO_2$: C, 66.36; H, 3.39; N, 3.36; found: C, 66.54; H, 3.20; N, 3.22.

2-Bromo-12-phenylbenzo[6,7]oxepino[3,4-*b*]quinolin-13(6*H*)-one (4h). Yellow solid, mp 179–180 °C; IR (KBr) ν/cm^{-1}: 3063, 1706, 1558, 1487, 1396, 1230, 1175, 1046, 909, 763, 716; ^{1}H NMR (600 MHz, $CDCl_3$) δ (ppm): 8.50 (d, *J* = 8.3 Hz, 1H, ArH), 8.19 (d, *J* = 8.4 Hz, 1H, ArH), 8.14 (d, *J* = 7.6 Hz, 1H, ArH), 7.85–7.87 (m, 1H, ArH), 7.77 (d, *J* = 7.3 Hz, 1H, ArH), 7.68–7.71 (m, 1H, ArH), 7.63 (td, *J* = 7.6, 1.0 Hz, 1H, ArH), 7.50 (t, *J* = 7.3 Hz, 1H, ArH), 7.37–7.40 (m, 2H, ArH), 6.99–7.01 (m, 2H, ArH), 5.70 (s, 2H, CH_2); ^{13}C NMR (150 MHz, $CDCl_3$) δ (ppm): 68.14, 113.30, 117.06, 122.88, 123.52, 124.56, 124.74, 124.82, 128.41, 131.14, 131.28, 132.19, 132.56, 133.90, 134.73, 142.09, 151.78, 152.59, 153.08, 158.12, 193.03; MS (ESI, *m/z*): 416.0 [M + H]$^+$; Anal. calcd for $C_{23}H_{14}BrNO_2$: C, 66.36; H, 3.39; N, 3.36; found: C, 66.17; H, 3.55; N, 3.22.

4-*tert*-Butyl-12-phenylbenzo[6,7]oxepino[3,4-*b*]quinolin-13(6*H*)-one (4i). White solid, mp 193–195 °C; IR (KBr) v/cm^{-1}: 3061, 2961, 2864, 1771, 1663, 1489, 1401, 1294, 1171, 1028, 1009, 766, 703; ^{1}H NMR (600 MHz, $CDCl_3$): δ 8.15 (d, *J* = 8.3 Hz, 1H, ArH), 7.72 (t, *J* = 7.7 Hz, 1H, ArH), 7.64 (d, *J* = 8.4 Hz, 1H, ArH), 7.54 (s, 1H, ArH), 7.40–7.47 (m, 5H, ArH), 7.16–7.18 (m, 2H, ArH), 6.93 (d, *J* = 8.6 Hz, 1H, ArH), 5.42 (s, 2H, CH_2); 1.22 (s, 9H, *tert*-butyl); ^{13}C NMR (150 MHz, $CDCl_3$): δ 31.30, 34.32, 75.48, 119.53, 121.98, 126.38, 126.75, 127.28, 127.51, 128.23, 128.35, 128.46, 129.25, 129.31, 129.58, 130.60, 130.86, 132.54, 133.61, 135.57, 144.82, 148.34, 148.54, 152.51, 157.58, 193.98; MS (ESI, *m/z*): 394.2 $[M + H]^+$; Anal. calcd for $C_{27}H_{23}NO_2$: C, 82.42; H, 5.89; N, 3.56; found: C, 82.21; H, 5.66; N, 3.72.

2-*tert*-Butyl−12-phenylbenzo[6,7]oxepino[3,4-*b*]quinolin-13(6*H*)-one (4j). Yellow crystals, mp 210–212 °C; IR (KBr) v/cm^{-1}: 3063, 2960, 2860, 1769, 1665, 1492, 1408, 1302, 1175, 1026, 1000, 766, 703; ^{1}H NMR (600 MHz, $CDCl_3$) δ (ppm): 8.13 (d, *J* = 8.4 Hz, 1H, ArH), 7.71 (t, *J* = 7.1 Hz, 1H, ArH), 7.63 (d, *J* = 8.4 Hz, 1H, ArH), 7.54 (d, *J* = 2.5 Hz, 1H, ArH), 7.39–7.47 (m, 5H, ArH), 7.16–7.18 (m, 2H, ArH), 6.93 (d, *J* = 8.6 Hz, 1H, ArH), 5.41 (s, 2H, CH_2), 1.22 (s, 9H, *tert*-butyl); ^{13}C NMR (150 MHz, $CDCl_3$) δ (ppm): 31.30, 34.32, 75.21, 114.60, 119.55, 126.15, 126.33, 126.75, 127.32, 127.63, 128.25, 128.53, 129.30, 131.05, 132.61, 133.63, 134.67, 135.47, 144.88, 152.39, 157.58, 193.80; MS (ESI, *m/z*): 394.2 $[M + H]^+$; Anal. calcd for $C_{27}H_{23}NO_2$: C, 82.42; H, 5.89; N, 3.56; found: C, 82.20; H, 5.67; N, 3.74.

2,4-Di-*tert*-butyl-12-phenylbenzo[6,7]oxepino[3,4-*b*]quinolin-13(6*H*)-one (4k). White crystals, mp 166–168 °C; IR (KBr) v/cm^{-1}: 3061, 2960, 2866, 1646, 1558, 1472, 1433, 1360, 1160, 1035, 1013, 911, 765, 700; ^{1}H NMR (600 MHz, $CDCl_3$) δ (ppm): 8.21 (d, *J* = 8.3 Hz, 1H, ArH), 7.79 (dd, *J* = 7.1, 8.2 Hz, 1H, ArH), 7.74 (d, *J* = 8.5 Hz, 1H, ArH), 7.46–7.58 (m, 6H, ArH), 7.28–7.30 (m, 2H, ArH), 5.51 (s, 2H, CH_2), 1.42 (s, 9H, *tert*-butyl), 1.30 (s, 9H, *tert*-butyl); ^{13}C NMR (150 MHz, $CDCl_3$) δ (ppm): 30.12, 31.39, 34.56, 35.59, 75.30, 125.32, 127.40, 127.46, 127.58, 128.25, 128.49, 129.43, 129.52, 130.08, 130.92, 133.70, 135.67, 139.82, 143.90, 153.25, 157.30, 194.32; MS (ESI, *m/z*): 450.2 $[M + H]^+$; Anal. calcd for $C_{31}H_{31}NO_2$: C, 82.82; H, 6.95; N, 3.12; found: C, 82.69; H, 6.64; N, 3.33.

2-*tert*-Butyl-4-chloro-12-phenylbenzo[6,7]oxepino[3,4-*b*]quinolin-13(6*H*)-one (4l). Yellow crystals. mp 179–180 °C; IR (KBr) v/cm^{-1}: 3066, 2958, 2867, 1712, 1603, 1560, 1505, 1396, 1261, 1060, 1007, 937, 764, 717; ^{1}H NMR (600 MHz, $CDCl_3$) δ (ppm): 8.38 (d, *J* = 8.4 Hz, 1H, ArH), 8.14 (d, *J* = 8.1 Hz, 1H, ArH), 8.03 (d, *J* = 7.6 Hz, 1H, ArH), 7.76 (t, *J* = 7.7 Hz, 1H, ArH), 7.58–7.65 (m, 2H, ArH), 7.53 (t, *J* = 7.6 Hz, 1H, ArH), 7.40 (t, *J* = 7.1 Hz, 1H, ArH), 7.31 (s, 1H, ArH), 7.16 (d, *J* = 8.6 Hz, 1H, ArH), 7.10 (d, *J* = 7.4 Hz, 1H, ArH), 5.68 (s, 2H, CH_2), 1.22 (s, 9H, *tert*-butyl); ^{13}C NMR (150 MHz, $CDCl_3$) δ (ppm): 31.35, 34.26, 69.11, 113.49, 113.67, 115.39, 120.66, 122.90, 123.41, 124.37, 124.67, 124.79, 128.42, 131.19, 132.51, 133.74, 134.68, 141.80, 145.29, 152.77, 153.60, 191.66; MS (ESI, *m/z*): 428.1 $[M + H]^+$; Anal. calcd for $C_{27}H_{22}ClNO_2$: C, 75.78; H, 5.18; N, 3.27; found: C, 75.57; H, 5.33; N, 3.43.

4-Chloro-12-phenylbenzo[6,7]oxepino[3,4-*b*]quinolin-13(6*H*)-one (4l'). Yellow solid. mp 200–202 °C; IR (KBr) v/cm^{-1}: 3291, 1663, 1570, 1491, 1405, 1301, 1183, 1098, 982, 742, 680; ^{1}H NMR (600 MHz, $CDCl_3$) δ (ppm): 8.34 (d, *J* = 8.4 Hz, 1H, ArH), 8.10 (d, *J* = 8.5 Hz, 1H, ArH), 7.99 (d, *J* = 7.6 Hz, 1H, ArH), 7.72–7.76 (m, 1H, ArH), 7.58 (dd, *J* = 7.2, 10.1 Hz, 2H, ArH), 7.51 (t, *J* = 7.6 Hz , 1H, ArH), 7.37 (t, *J* = 7.4 Hz, 1H, ArH), 7.18 (d, *J* = 8.2 Hz, 1H, ArH), 7.00–7.04 (m, 2H, ArH), 6.84–6.89 (m, 1H, ArH), 5.68 (s, 2H, CH_2); ^{13}C NMR (150 MHz, $CDCl_3$) δ (ppm): 68.75, 115.84, 116.14, 116.32, 121.56, 121.63, 122.92, 123.42, 124.26, 124.29, 124.42, 124.68, 124.85, 128.54, 130.68, 131.30, 132.65, 133.74, 134.74, 141.74, 147.00, 147.11, 151.62, 152.43, 154.07, 192.61; MS (ESI, *m/z*): 372.1 $[M + H]^+$; Anal. calcd for $C_{23}H_{14}ClNO_2$: C, 74.30; H, 3.80; N, 3.77; found: C, 74.14; H, 3.98; N, 3.63.

^{1}H and ^{13}C NMR spectra of compounds 3a–l

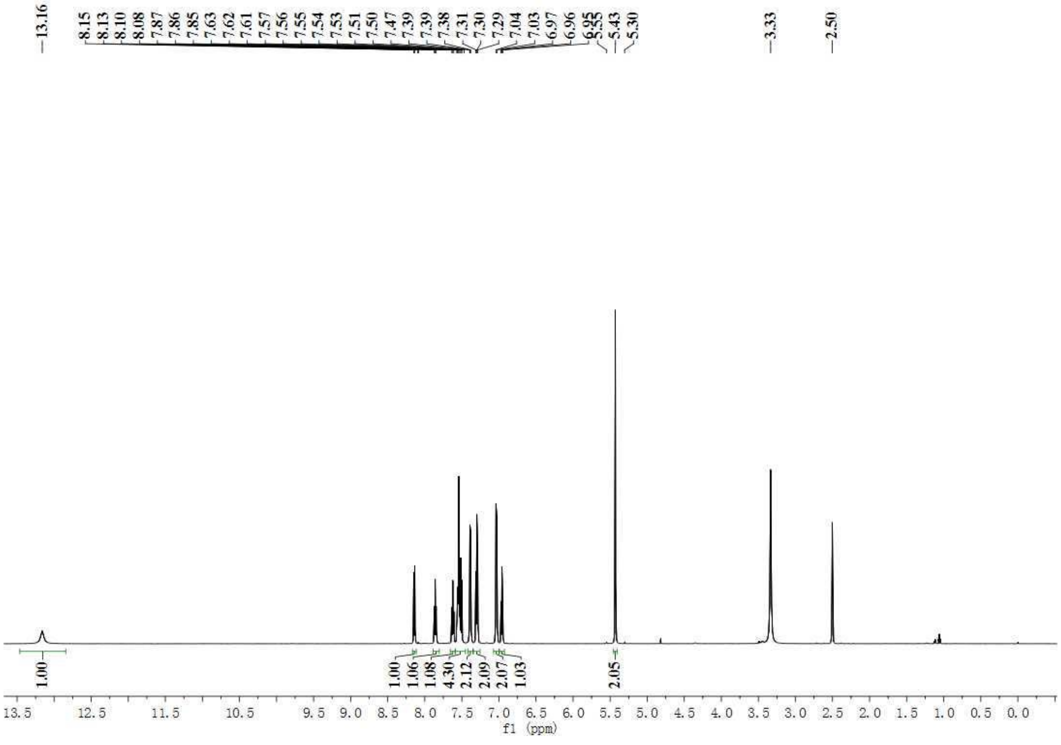

Figure S1. ^{1}H NMR spectrum of **3a**.

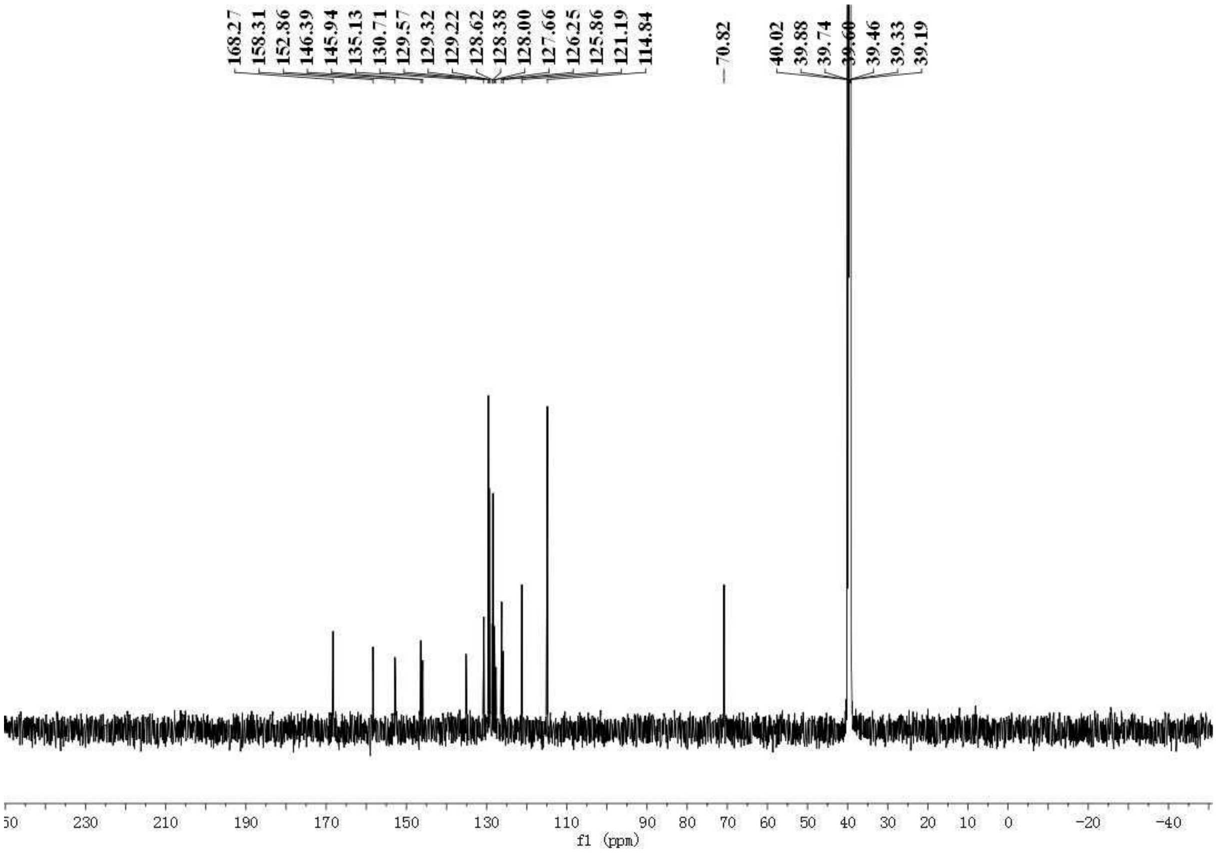

Figure S2. ^{13}C NMR spectrum of **3a**.

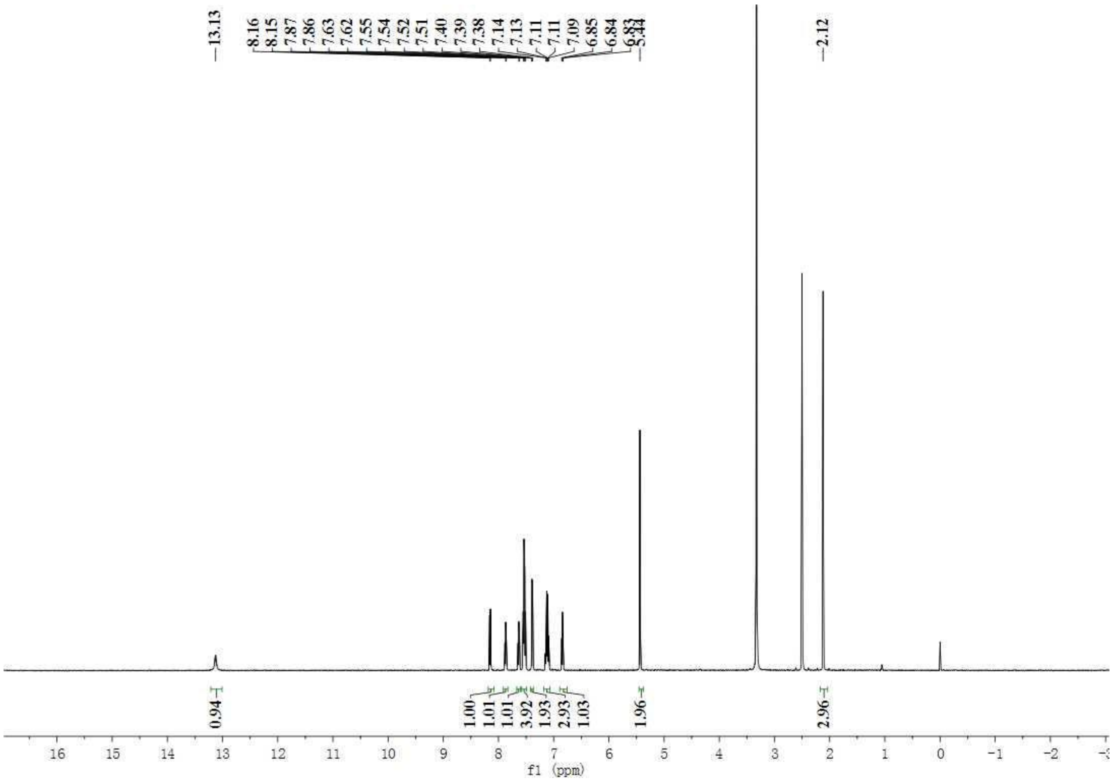

Figure S3. ^{1}H NMR spectrum of **3b**.

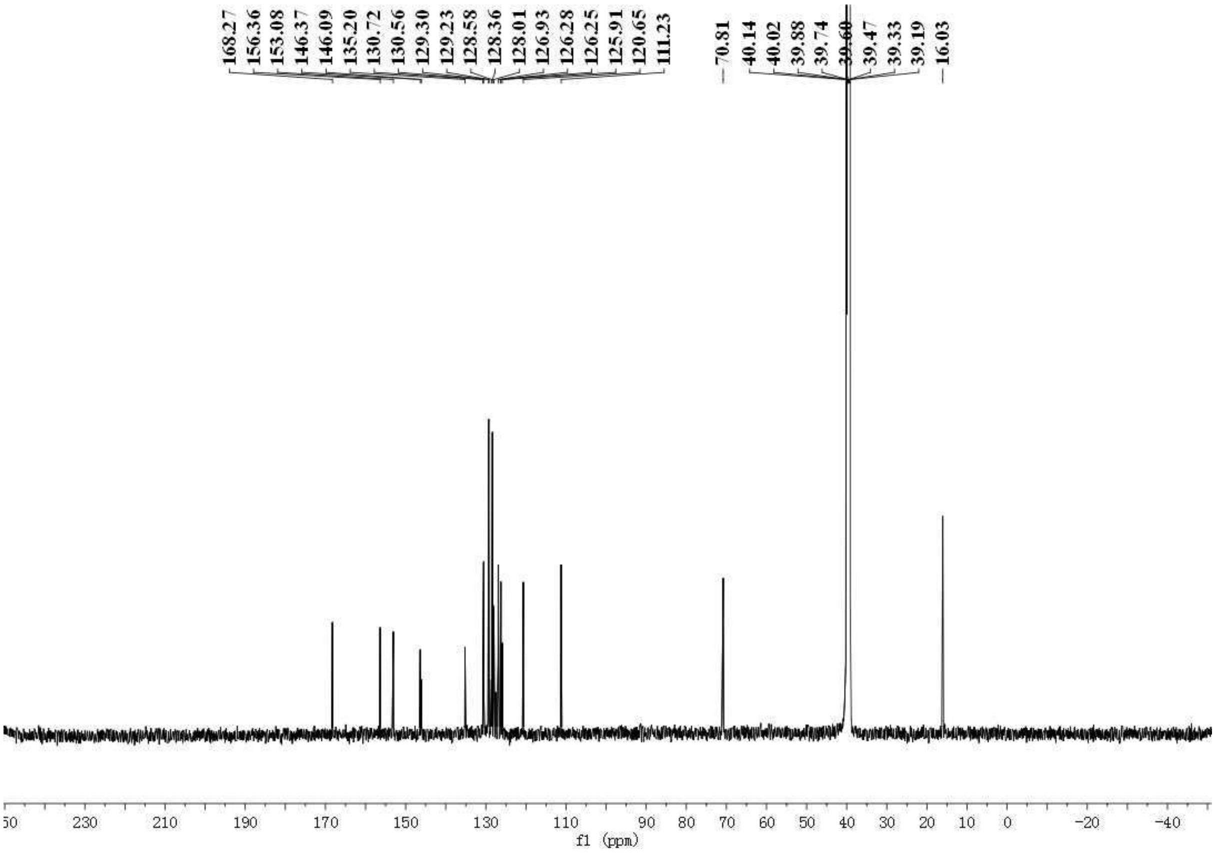

Figure S4. ^{13}C NMR spectrum of **3b**.

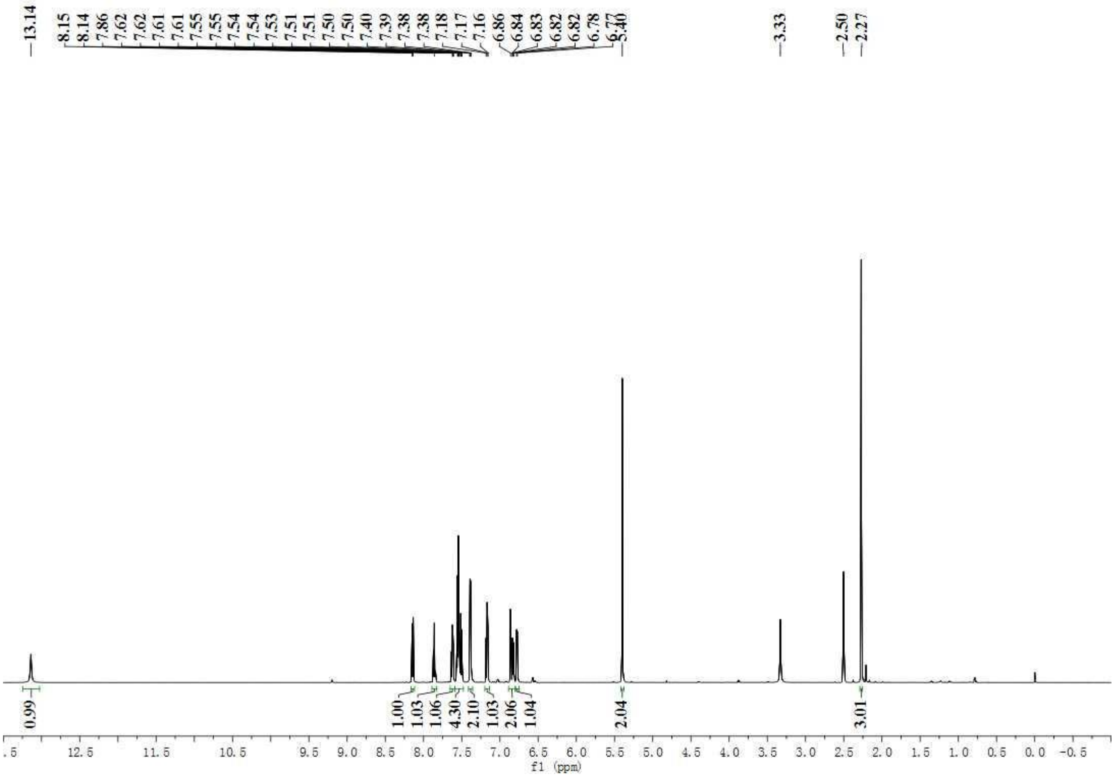

Figure S5. ^{1}H NMR spectrum of **3c**.

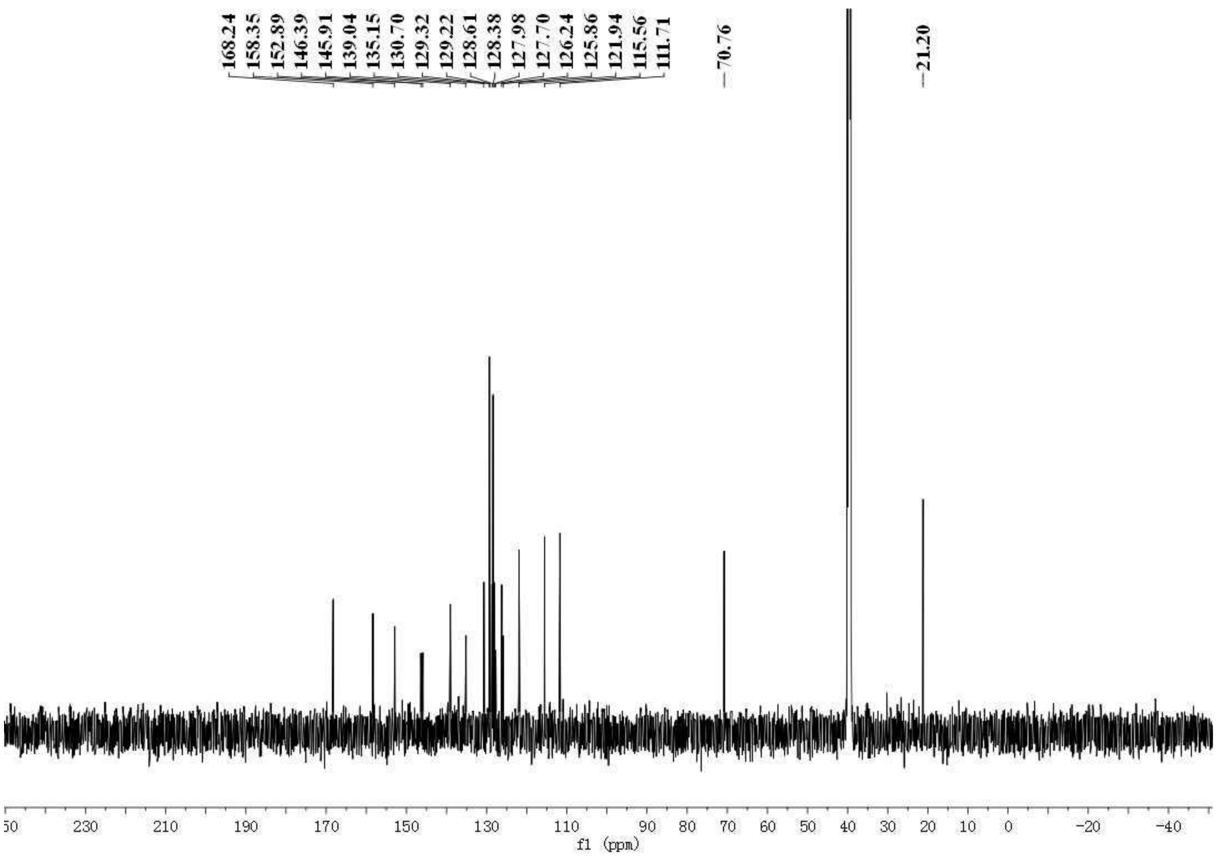

Figure S6. ^{13}C NMR spectrum of **3c**.

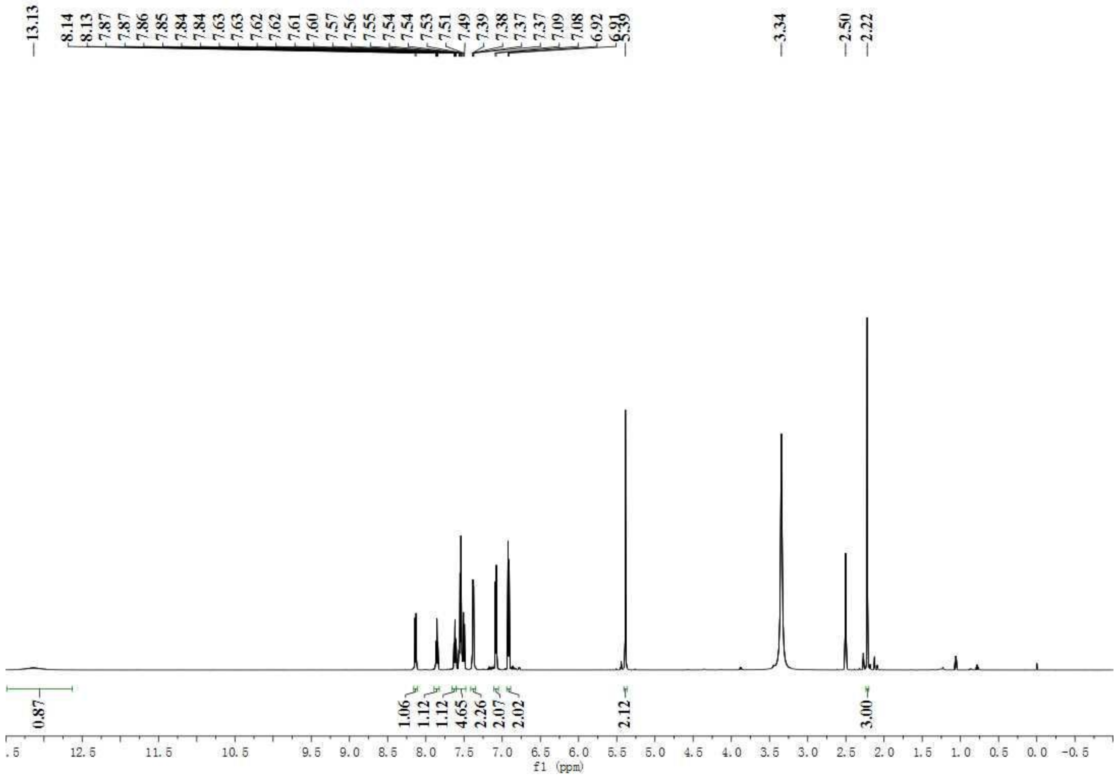

Figure S7. ^{1}H NMR spectrum of **3d**.

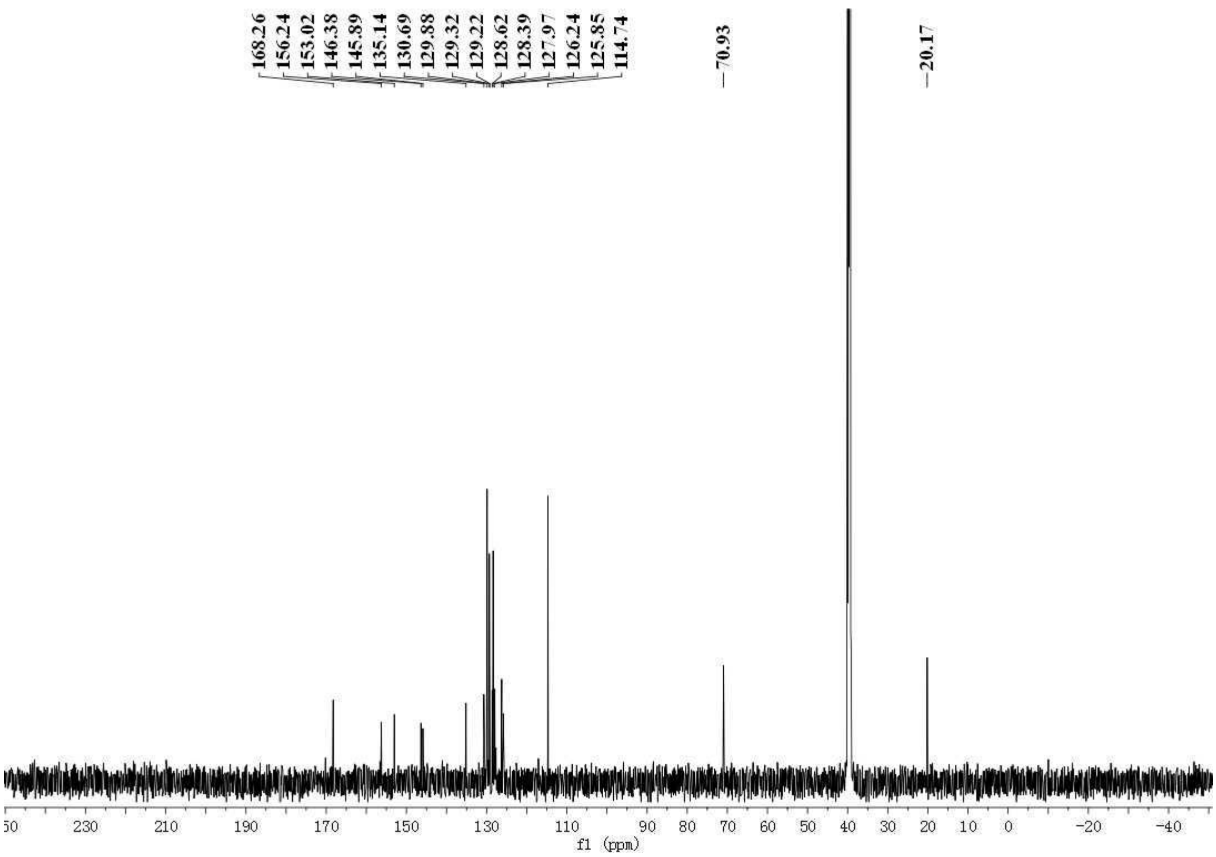

Figure S8. ^{13}C NMR spectrum of **3d**.

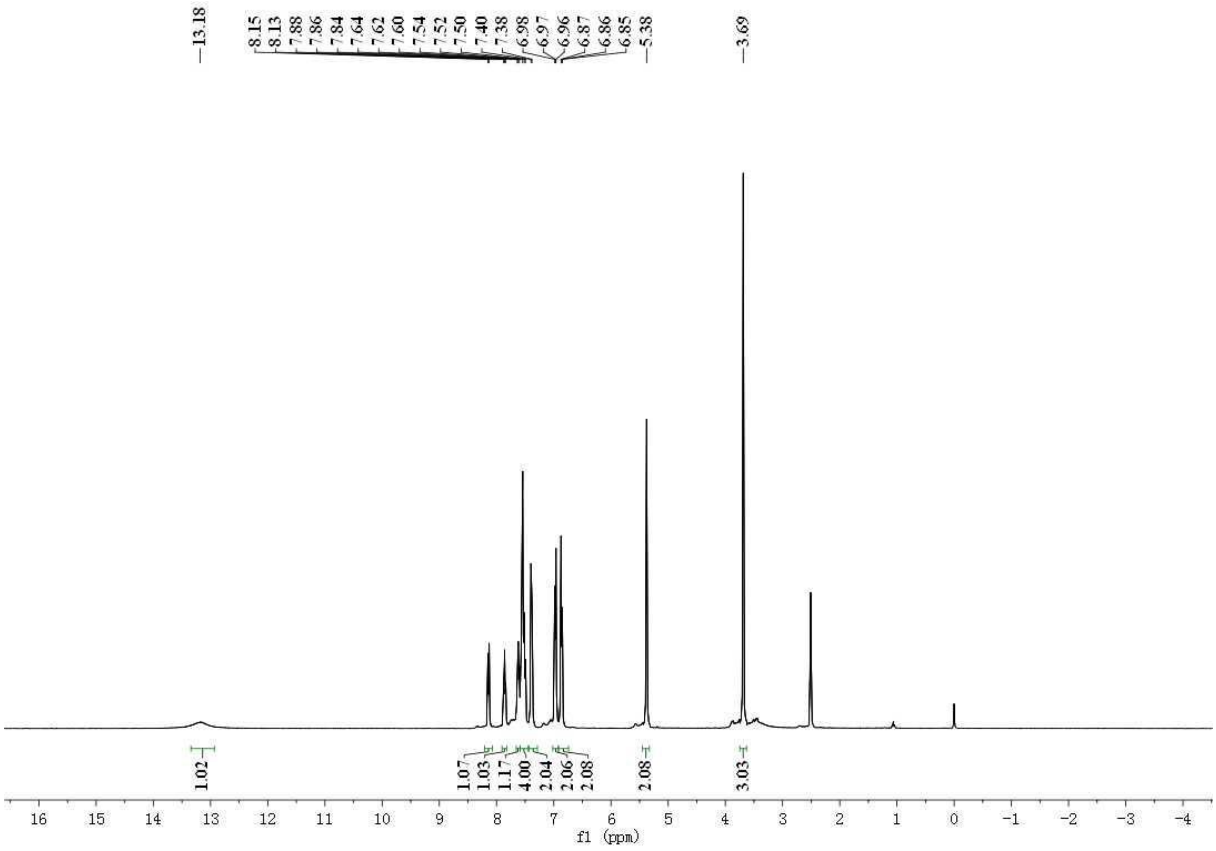

Figure S9. ^{1}H NMR spectrum of **3e**.

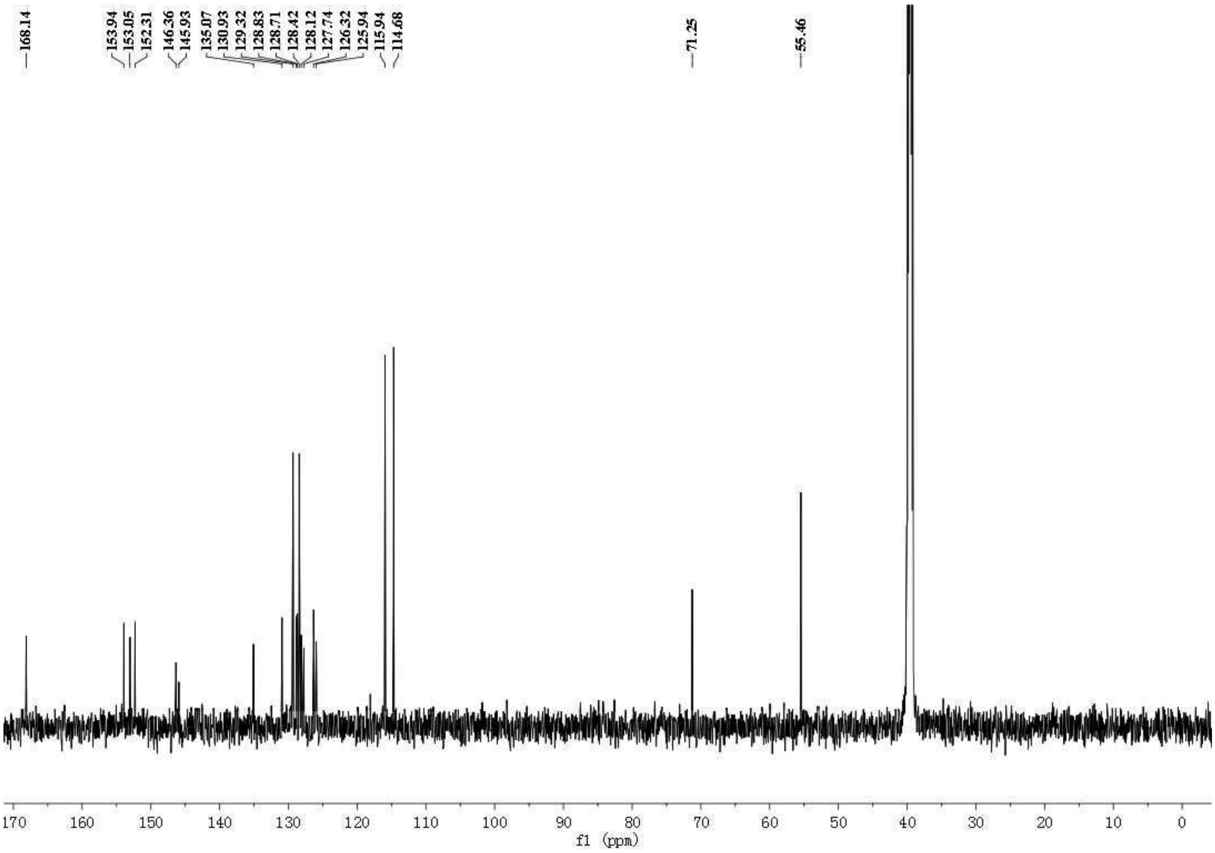

Figure S10. ^{13}C NMR spectrum of **3e**.

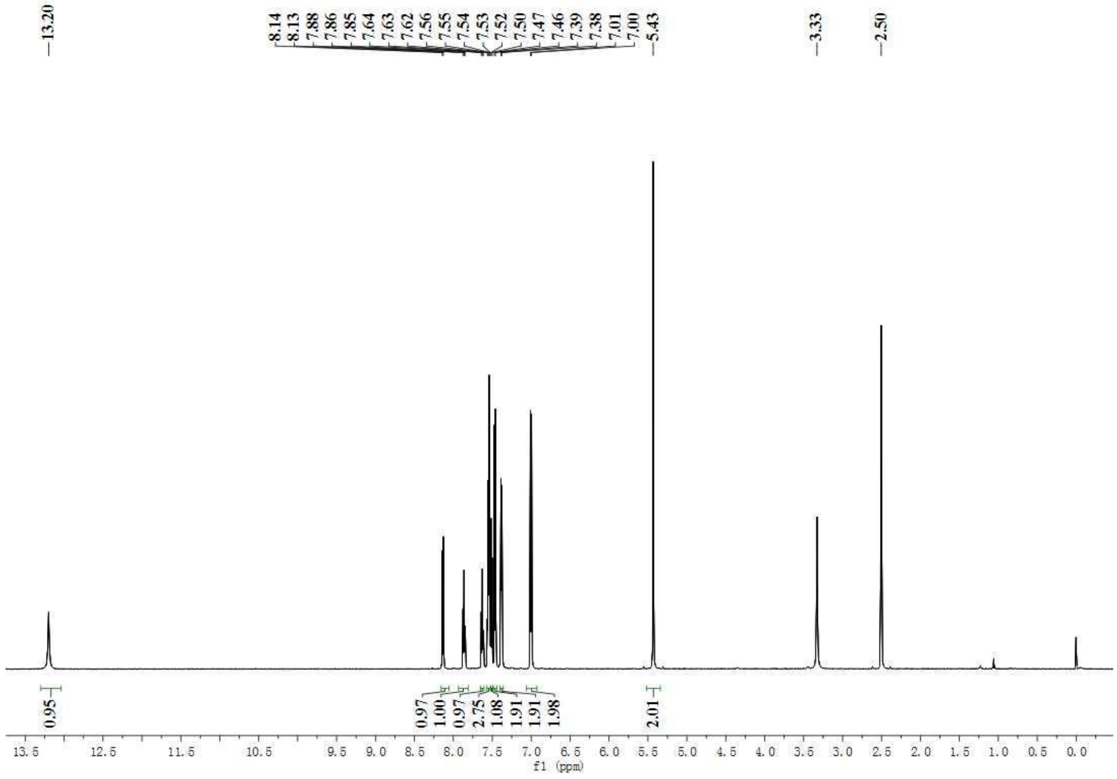

Figure S11. ^{1}H NMR spectrum of **3f**.

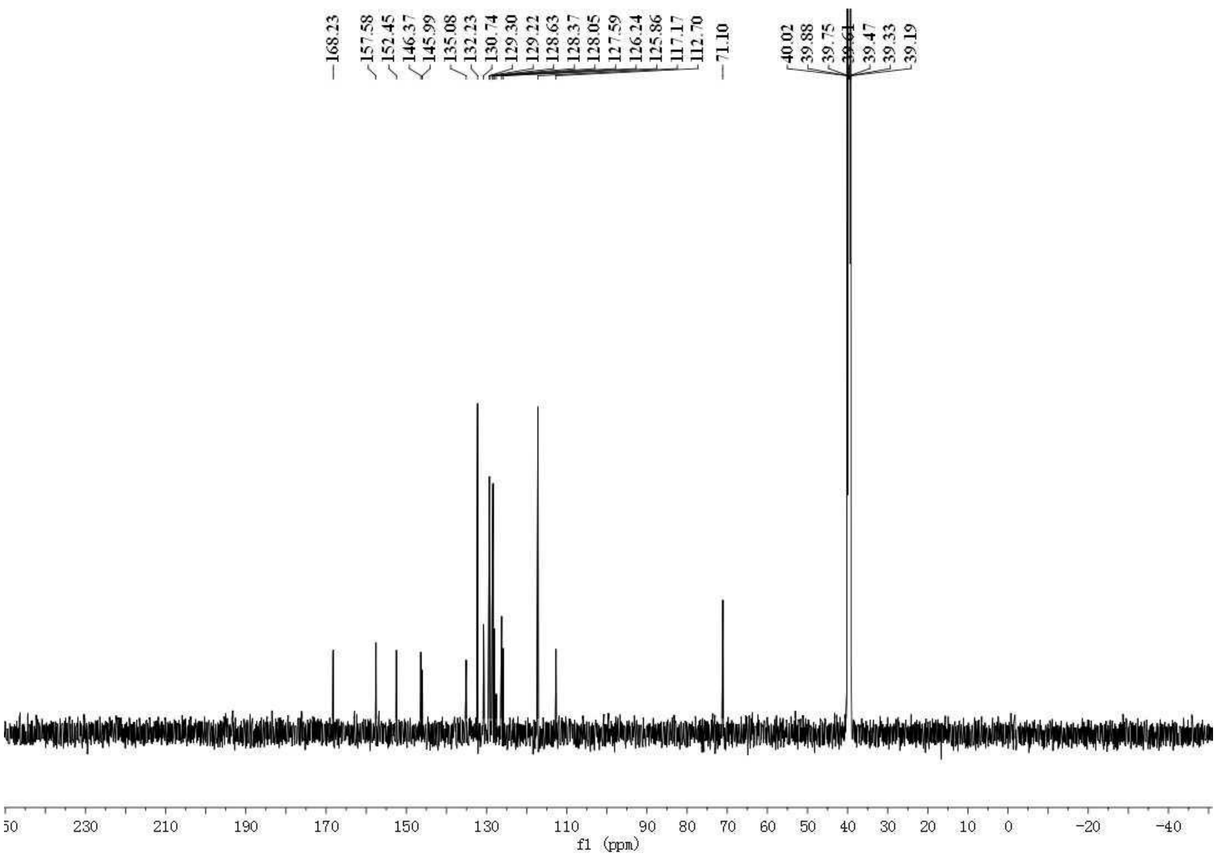

Figure S12. ^{13}C NMR spectrum of **3f**.

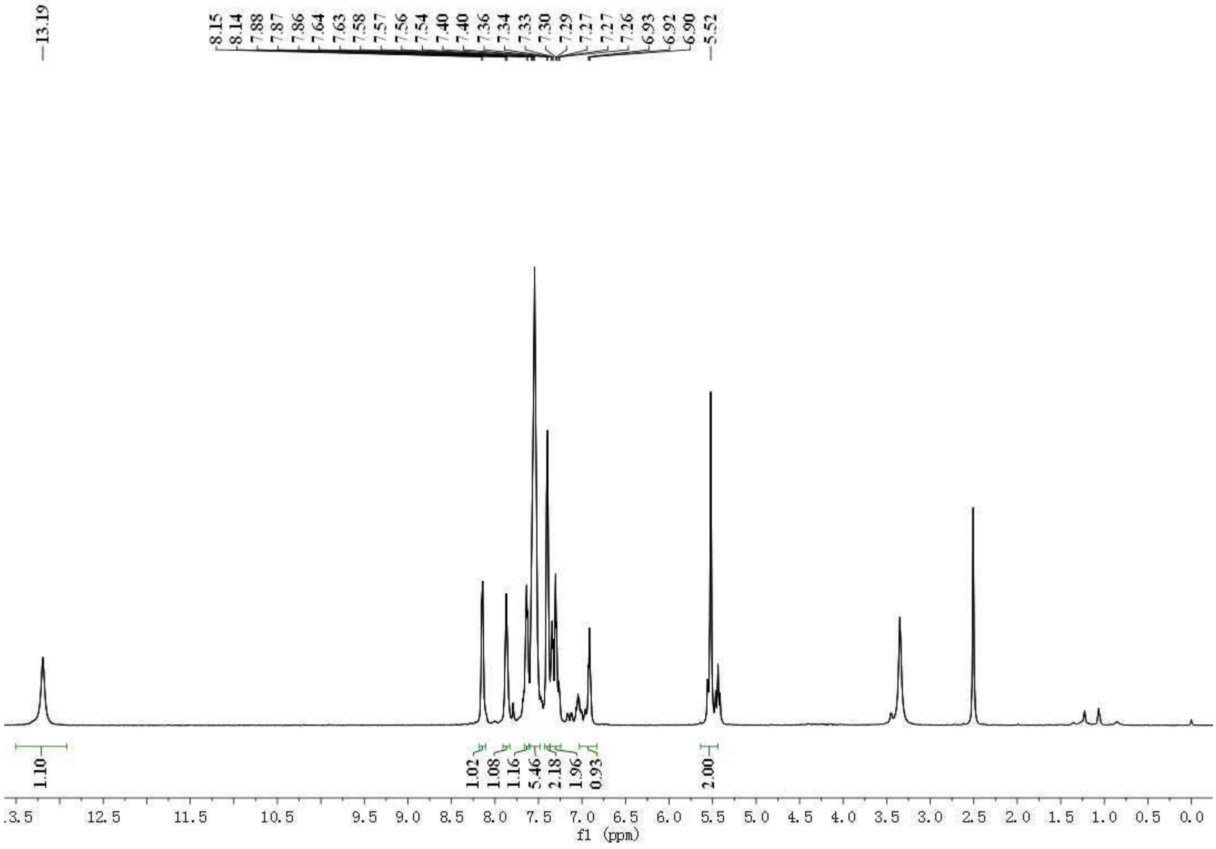

Figure S13. ^{1}H NMR spectrum of **3g**.

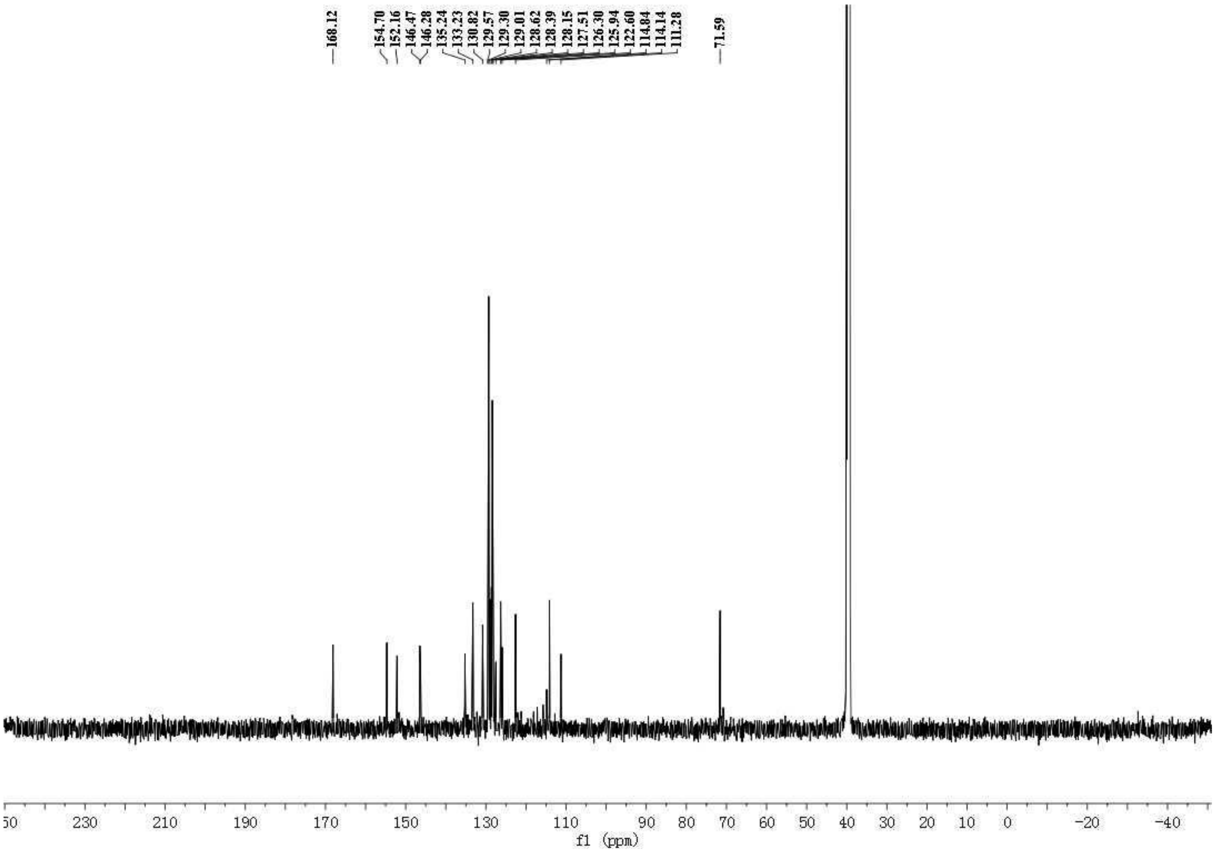

.**Figure S14.** ^{13}C NMR spectrum of **3g**.

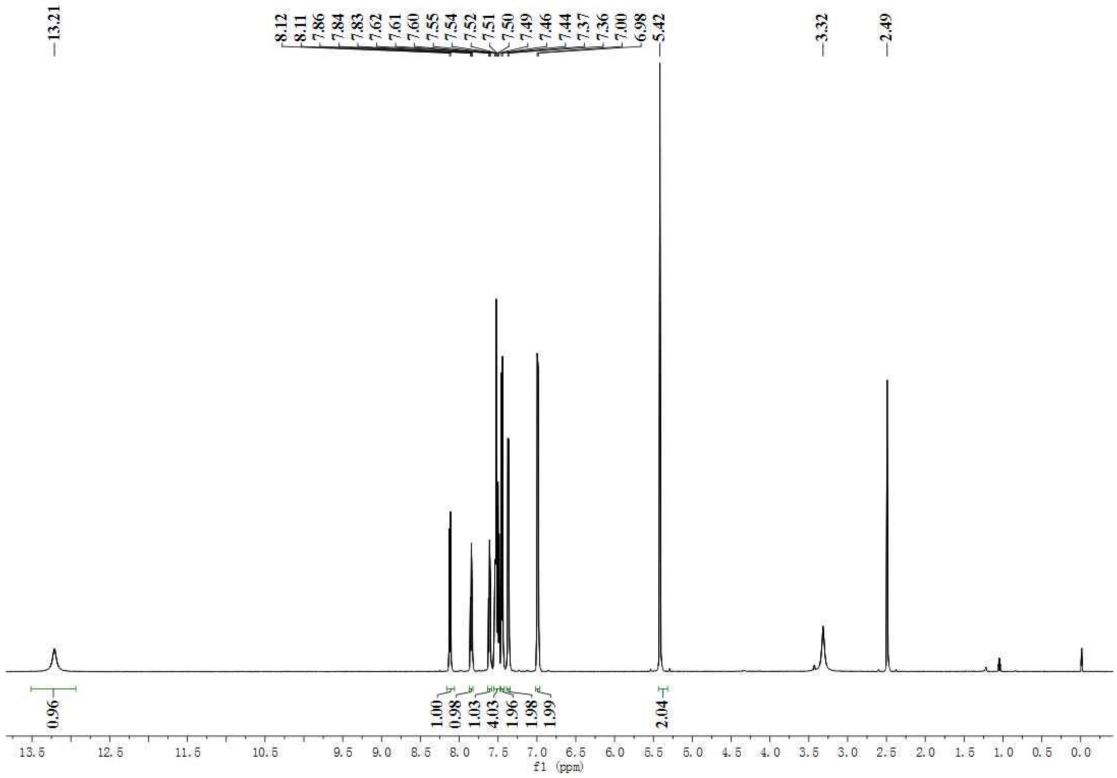

Figure S15. ^{1}H NMR spectrum of **3h**.

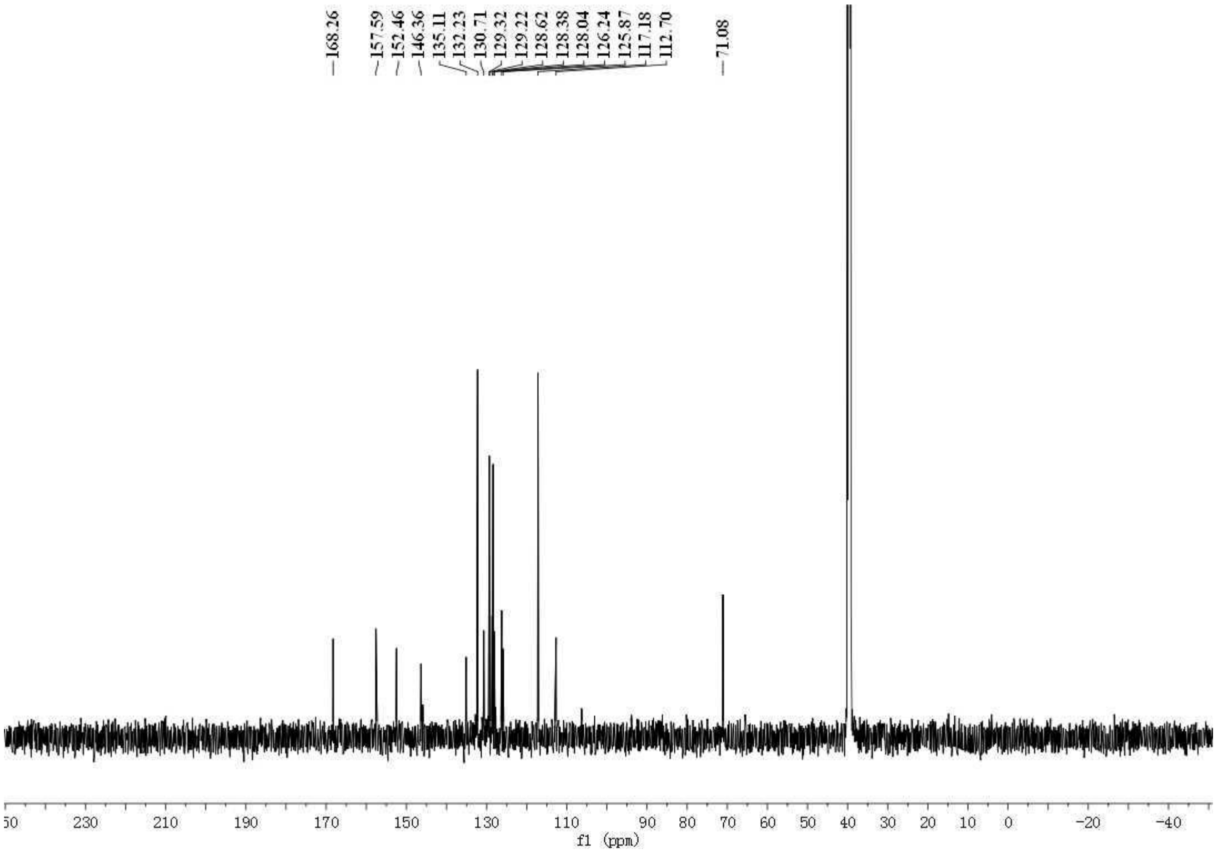

Figure S16. ^{13}C NMR spectrum of **3h**.

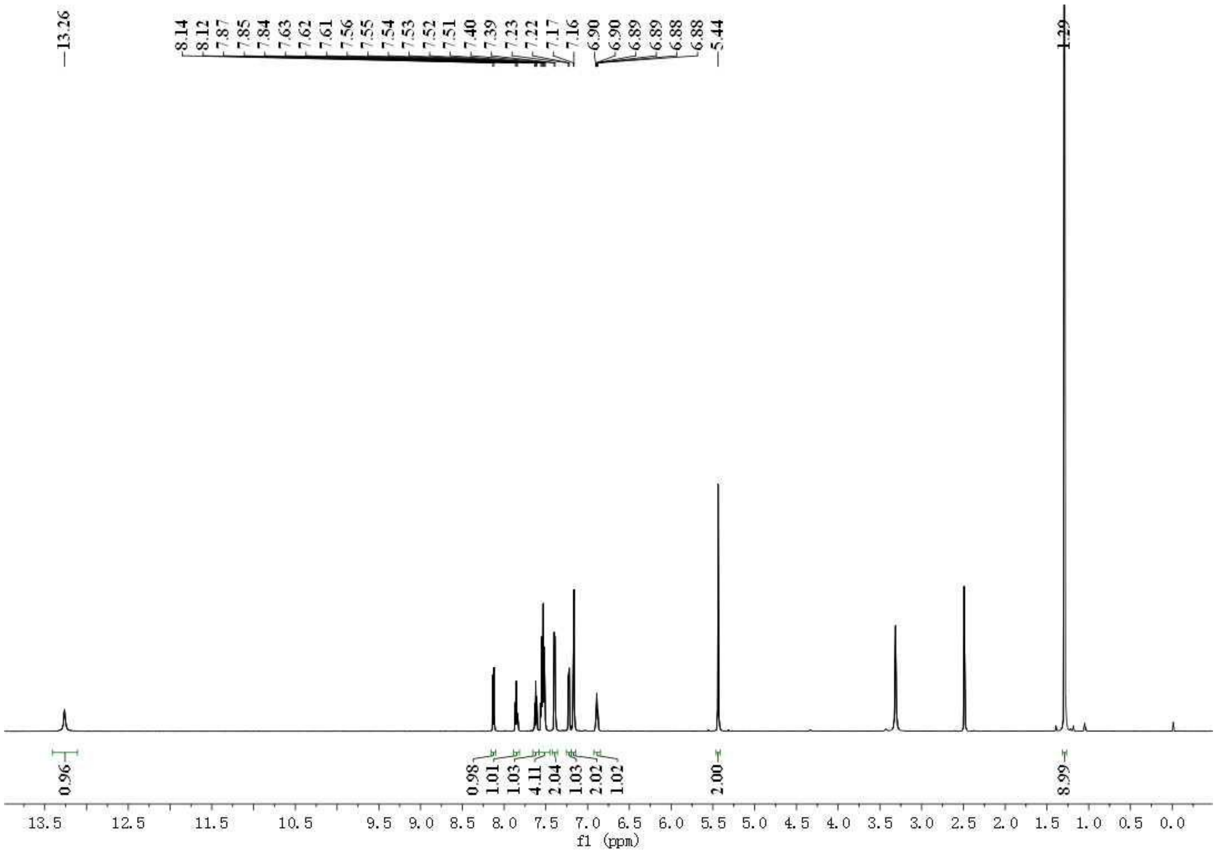

Figure S17. ^{1}H NMR spectrum of **3i**.

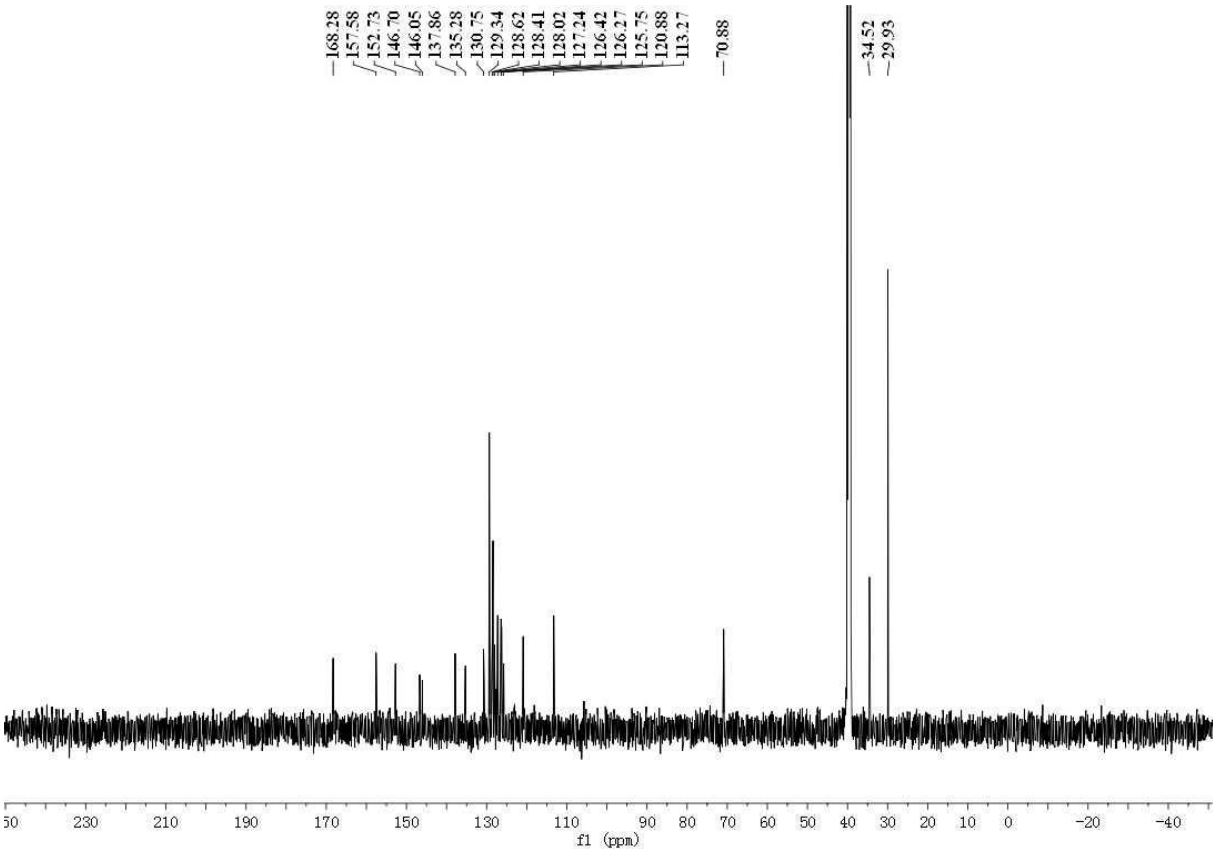

Figure S18. ^{13}C NMR spectrum of **3i**.

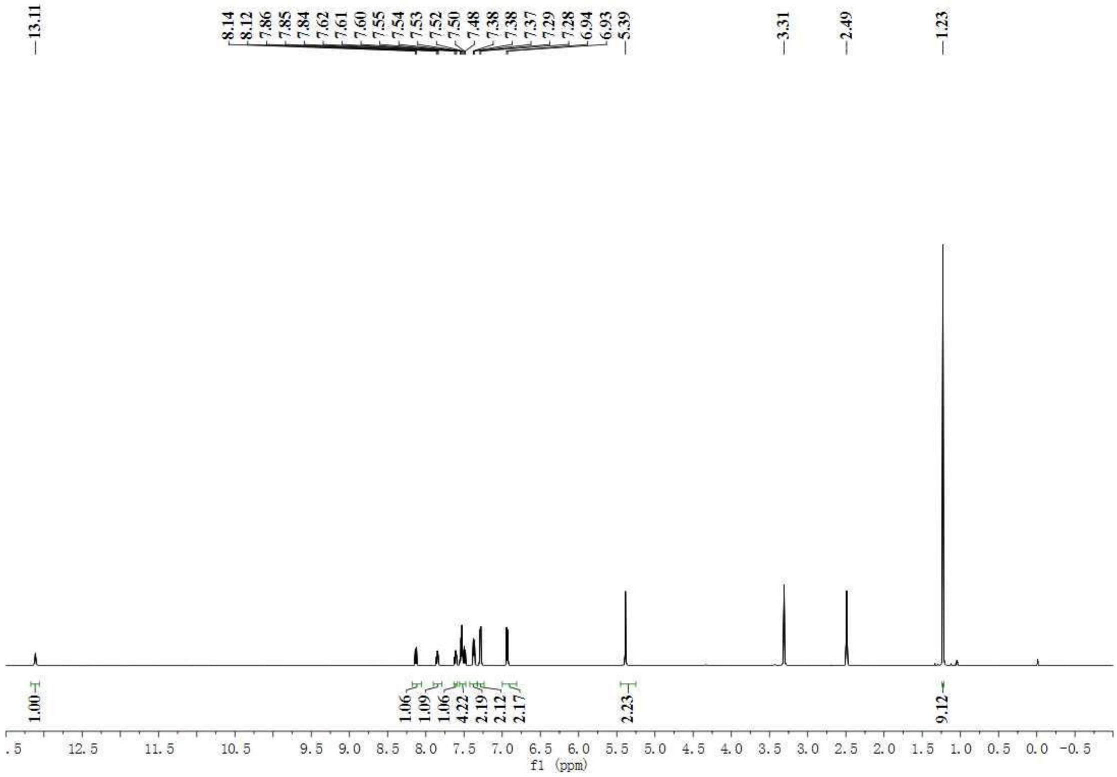

Figure S19. ^{1}H NMR spectrum of **3j**.

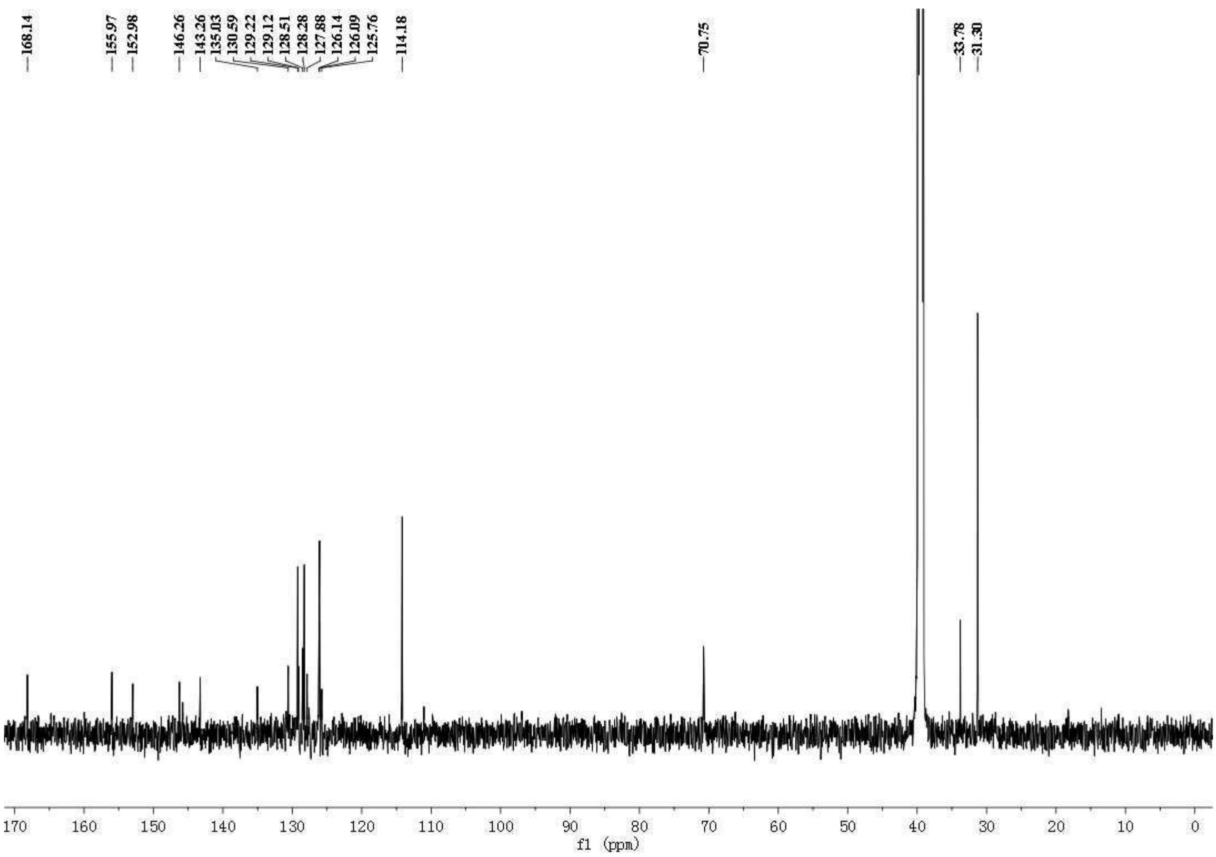

Figure S20. ^{13}C NMR spectrum of **3j**.

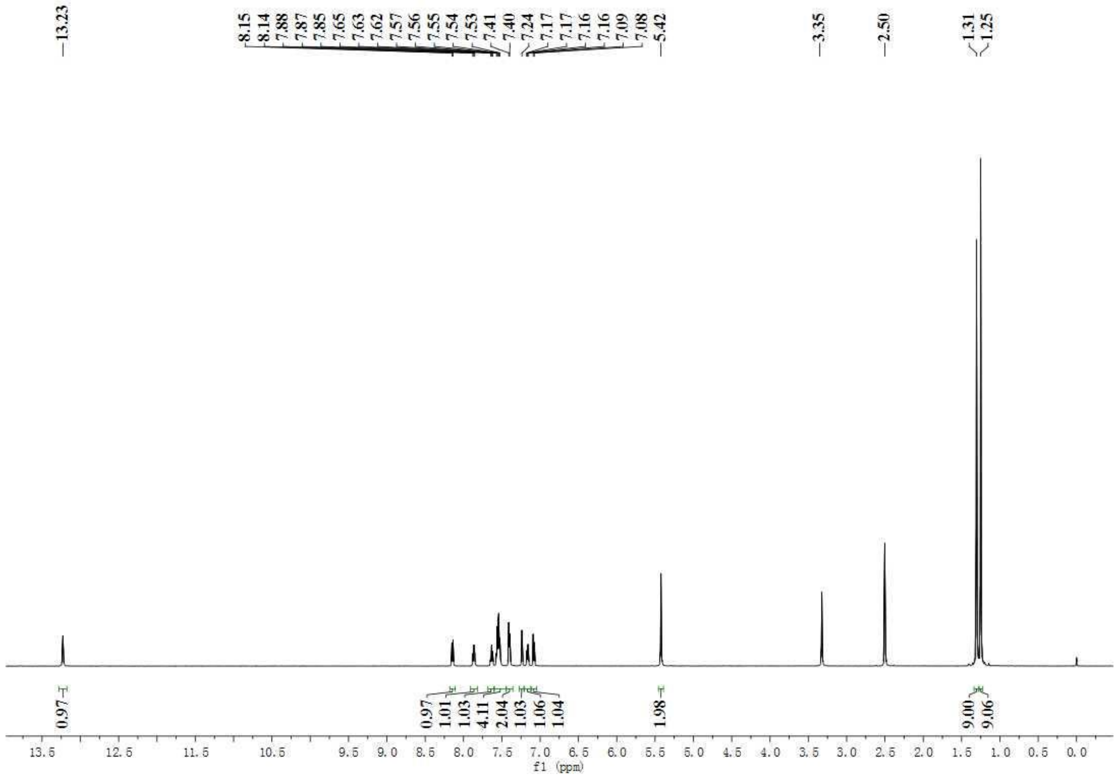

Figure S21. ^{1}H NMR spectrum of **3k**.

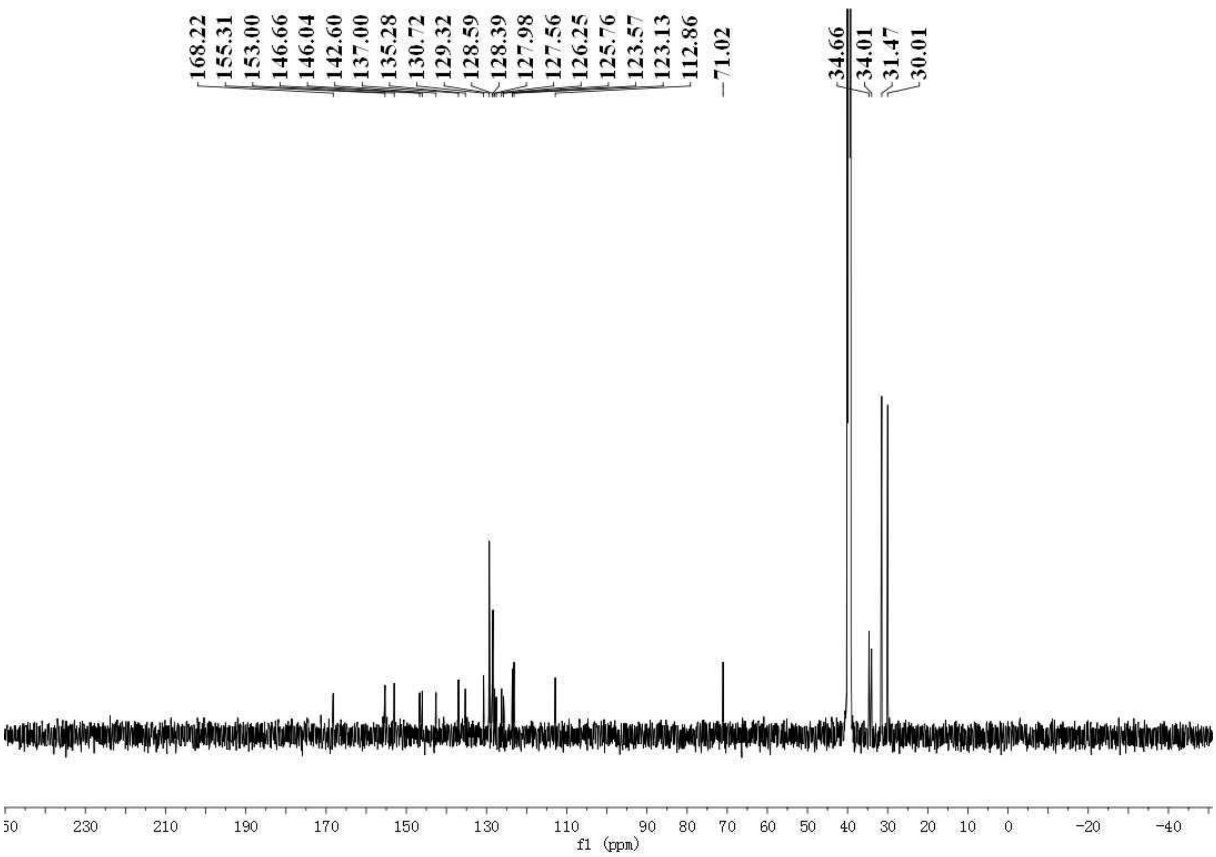

Figure S22. ^{13}C NMR spectrum of **3k**.

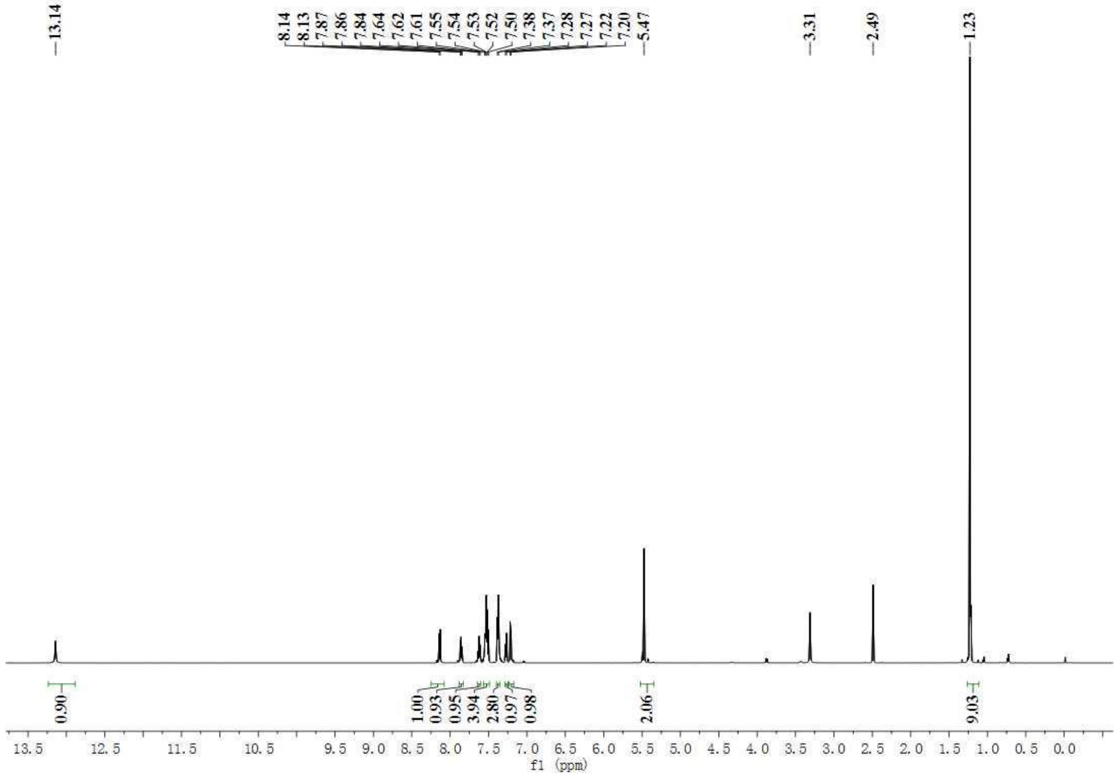

Figure S23. ^{1}H NMR spectrum of **3l**.

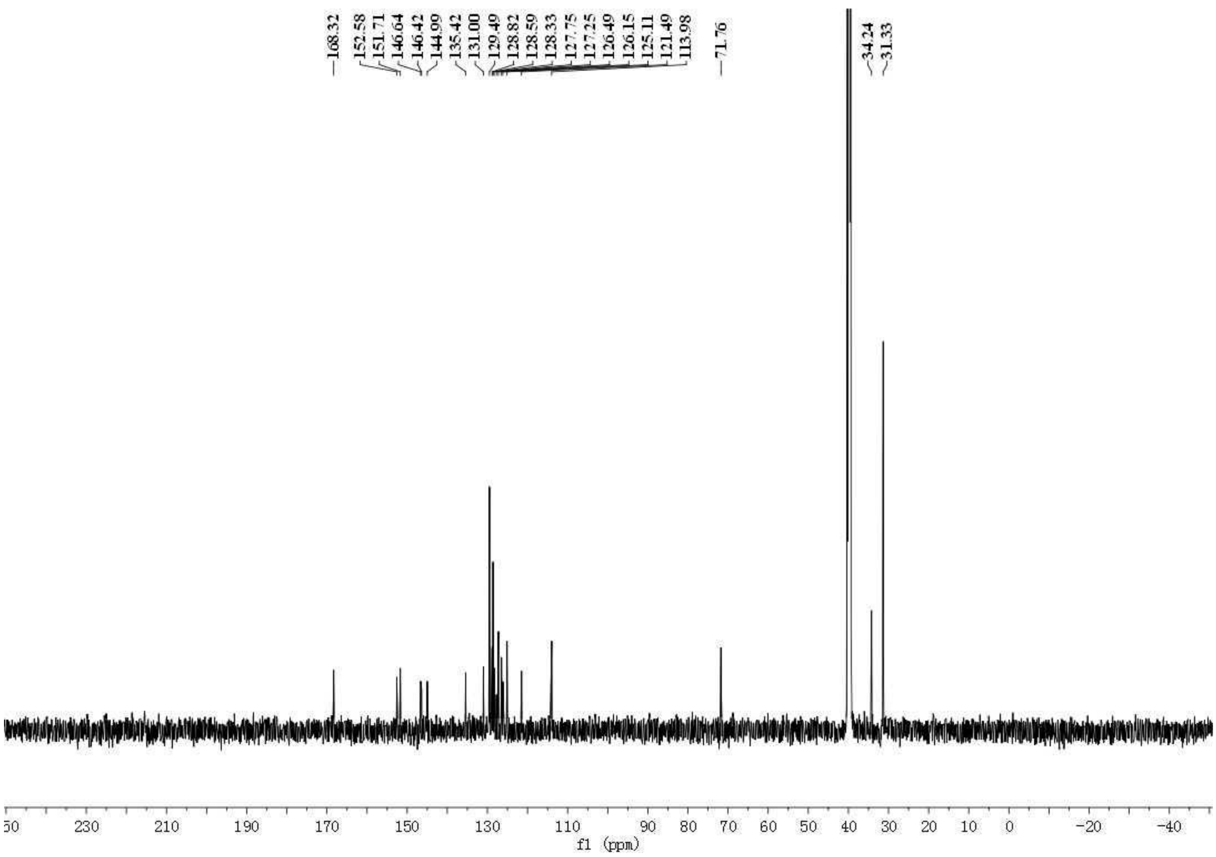

Figure S24. ^{13}C NMR spectrum of **3l**.

^{1}H and ^{13}C NMR spectra of compounds 4a–l

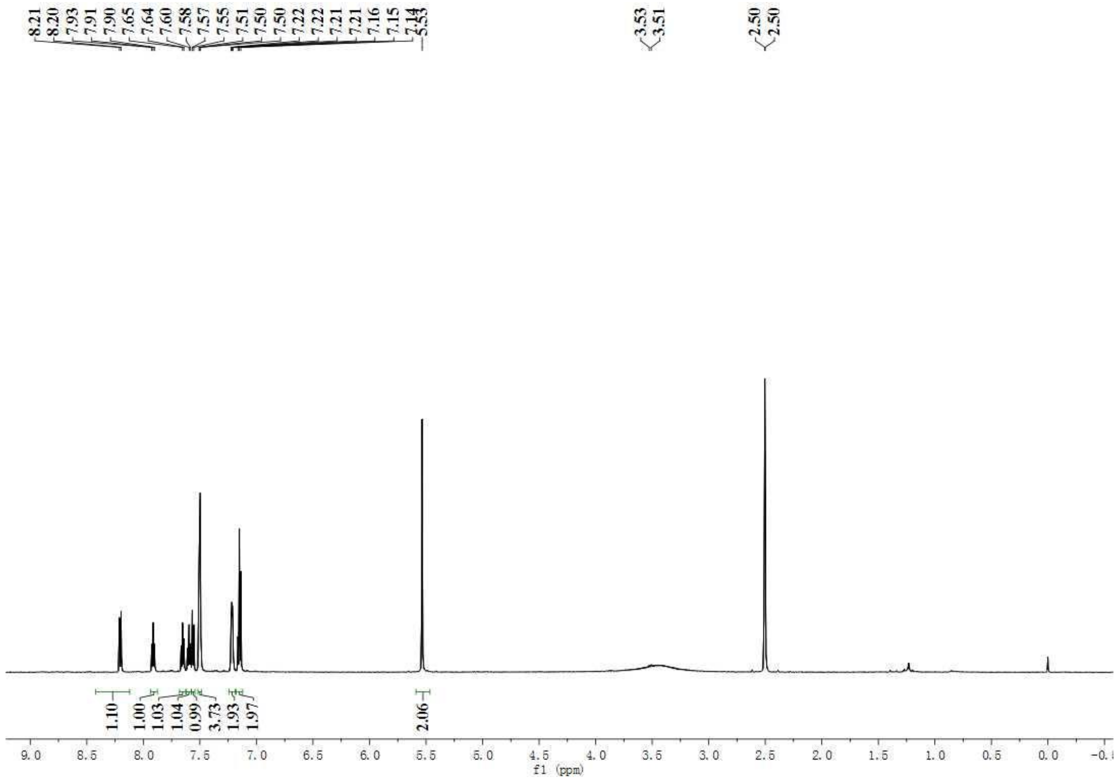

Figure S25. ^{1}H NMR spectrum of **4a**.

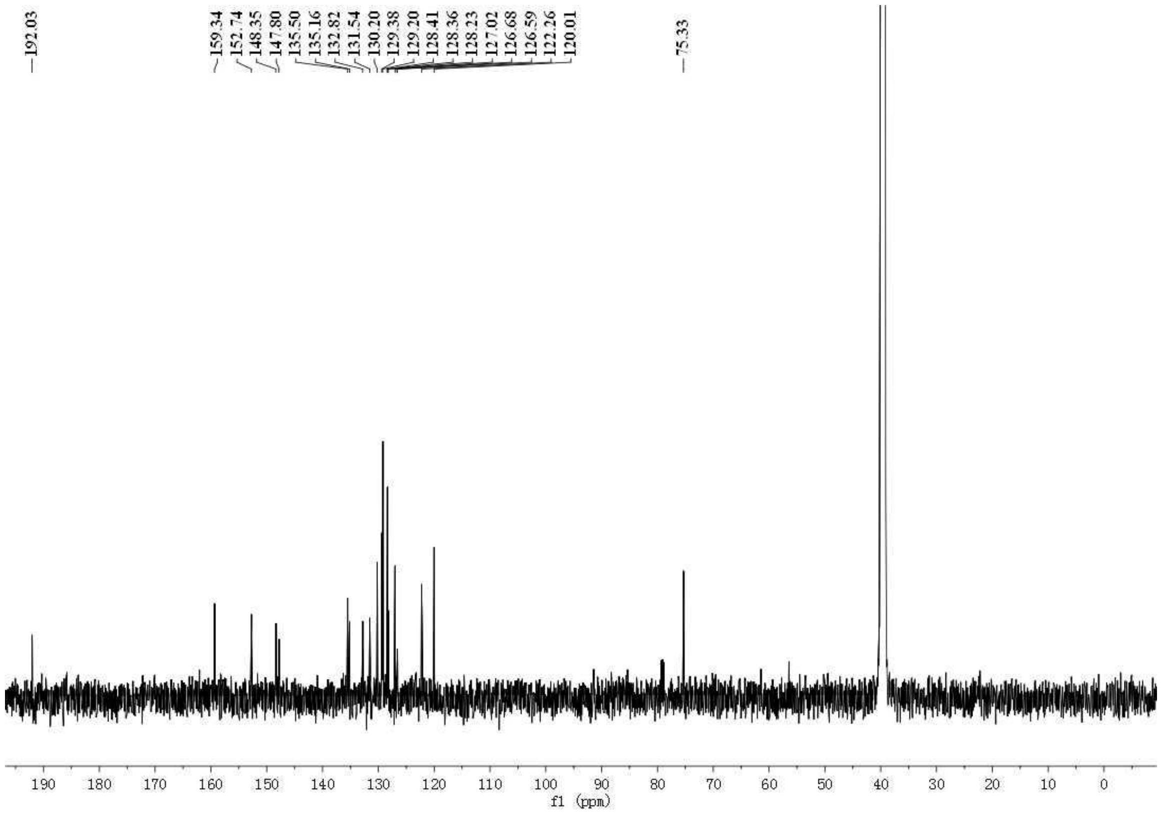

Figure S26. ^{13}C NMR spectrum of **4a**.

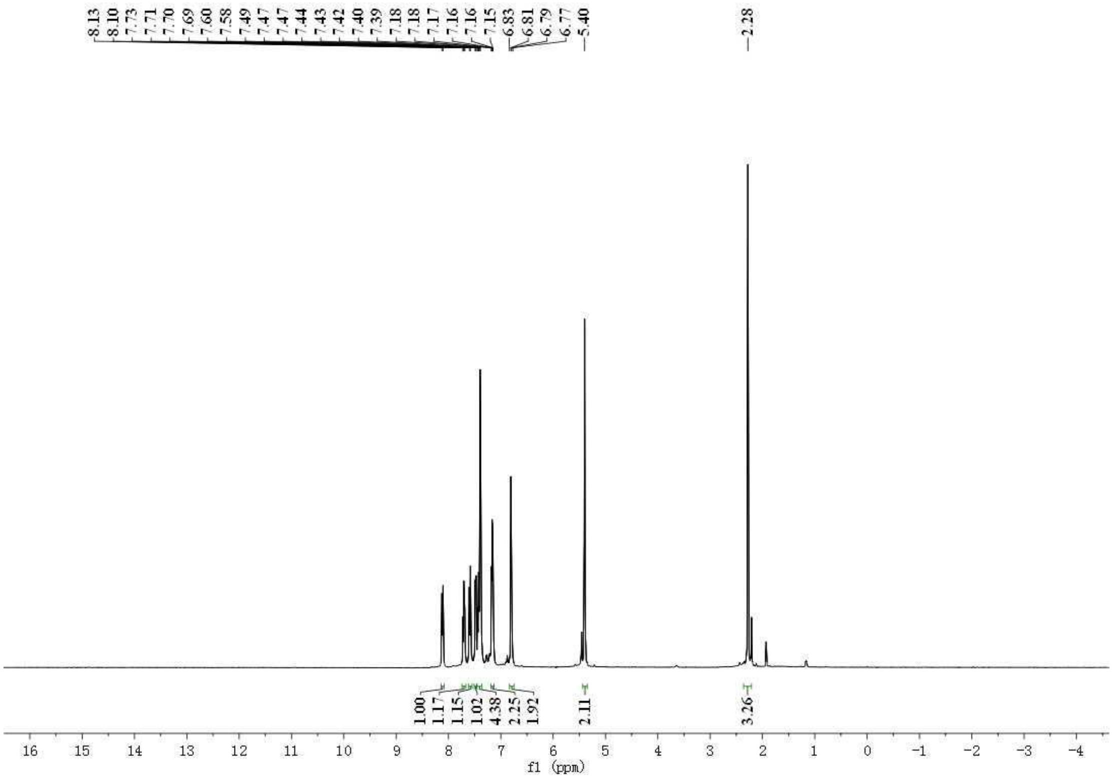

Figure S27. ^{1}H NMR spectrum of **4b**.

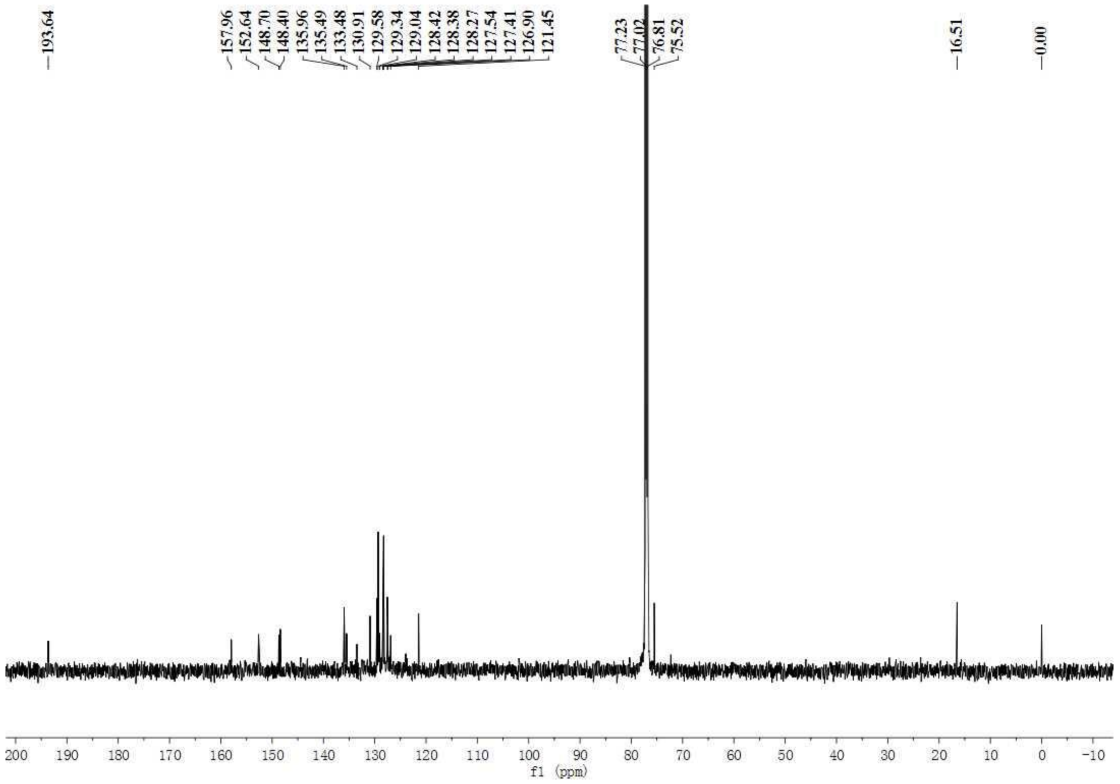

Figure S28. ^{13}C NMR spectrum of **4b**.

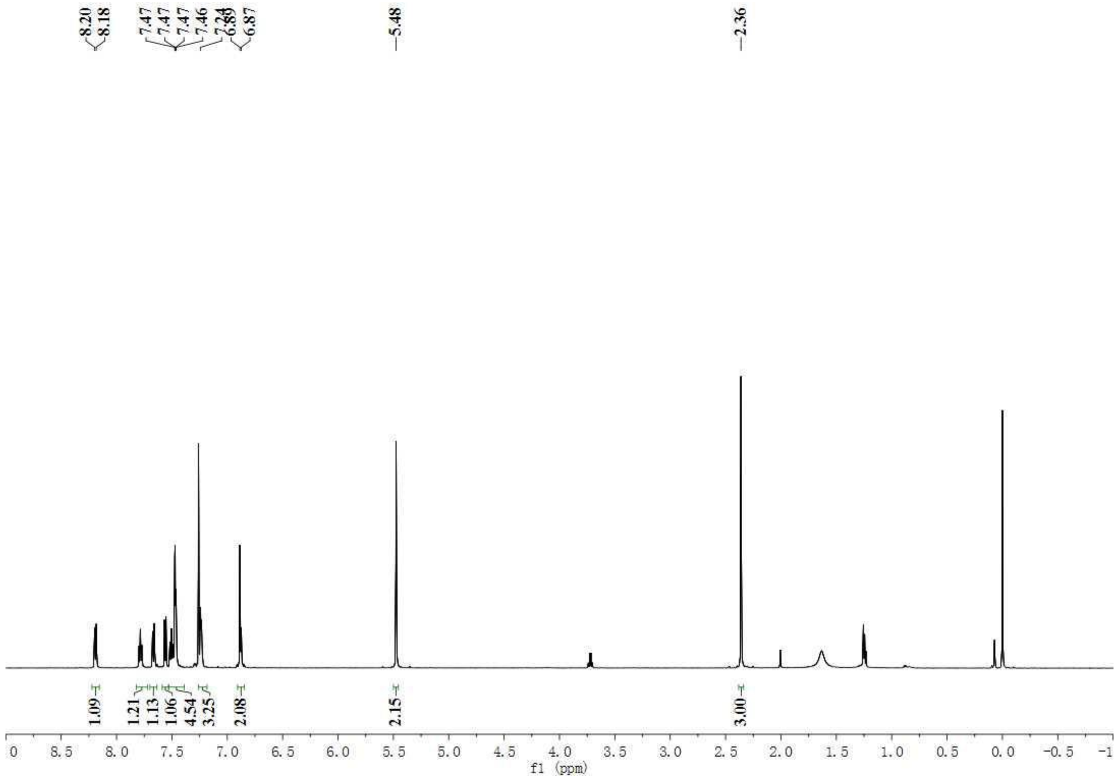

Figure S29. ^{1}H NMR spectrum of **4c**.

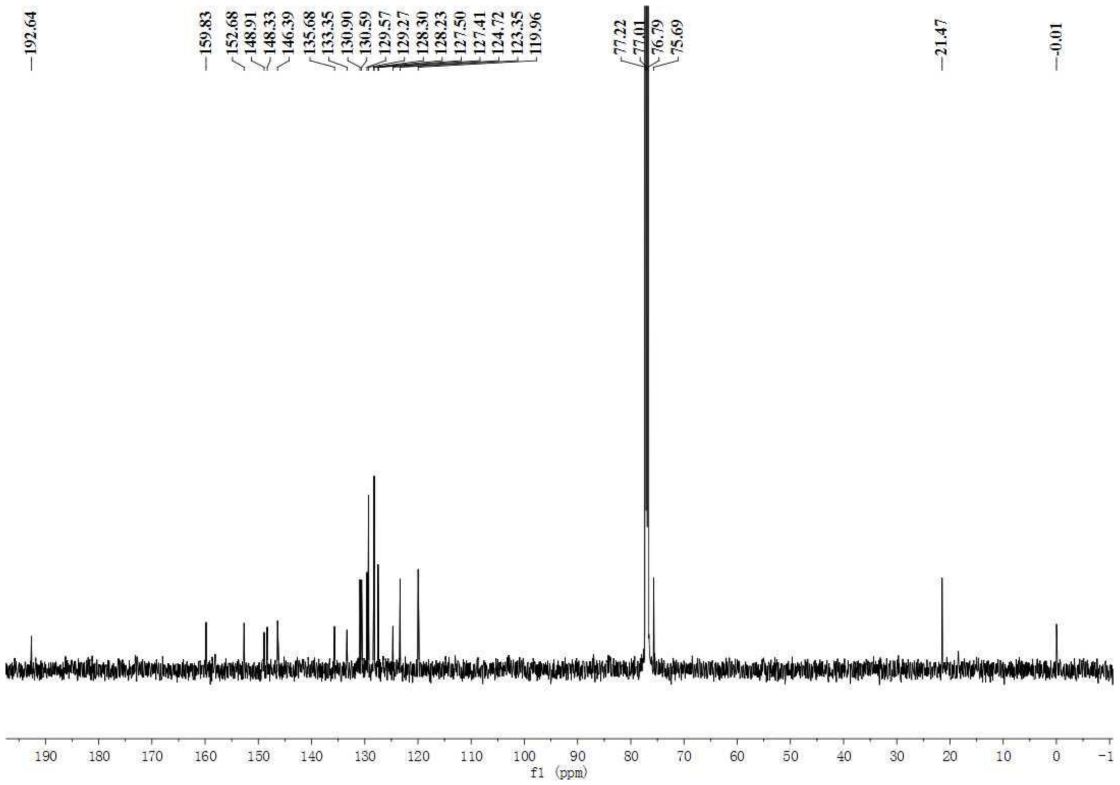

Figure S30. ^{13}C NMR spectrum of **4c**.

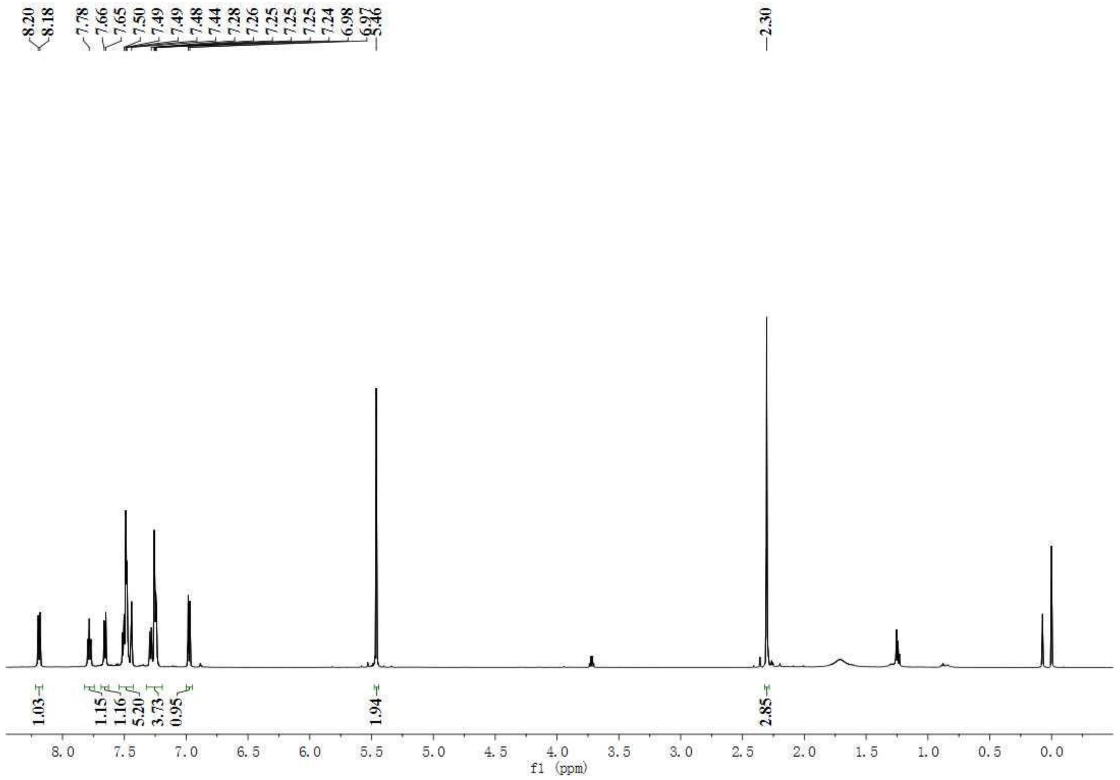

Figure S31. ^{1}H NMR spectrum of **4d**.

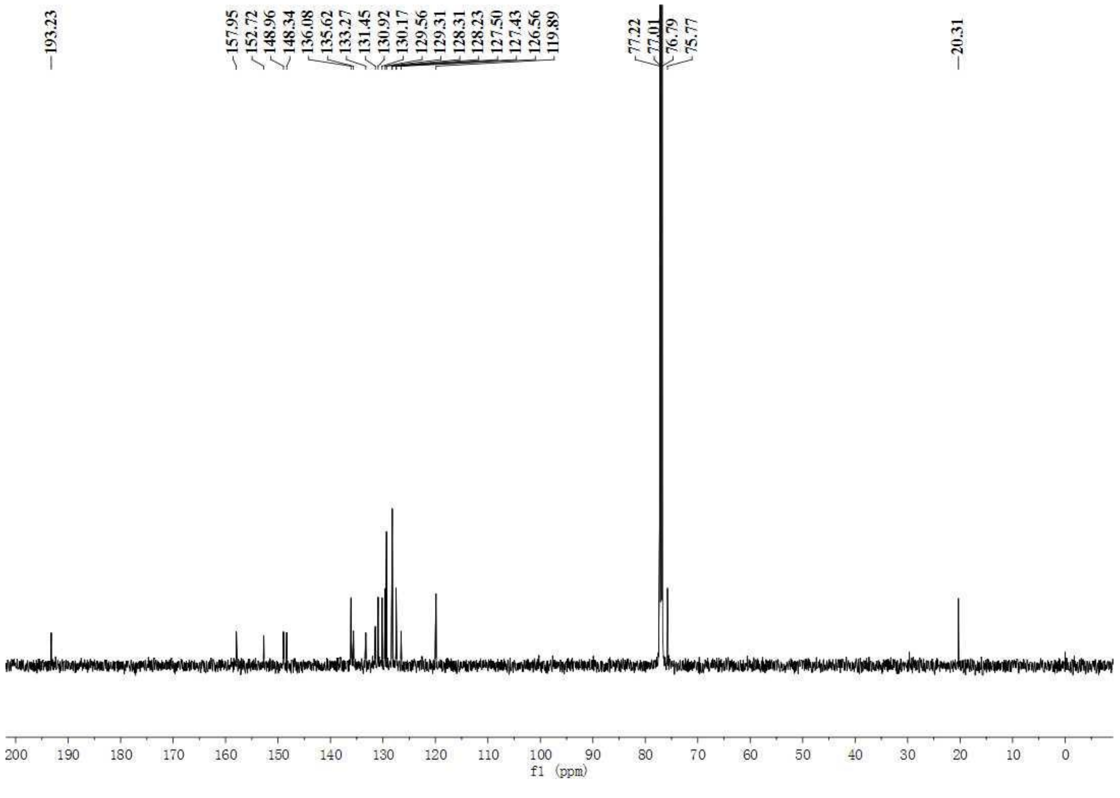

Figure S32. ^{13}C NMR spectrum of **4d**.

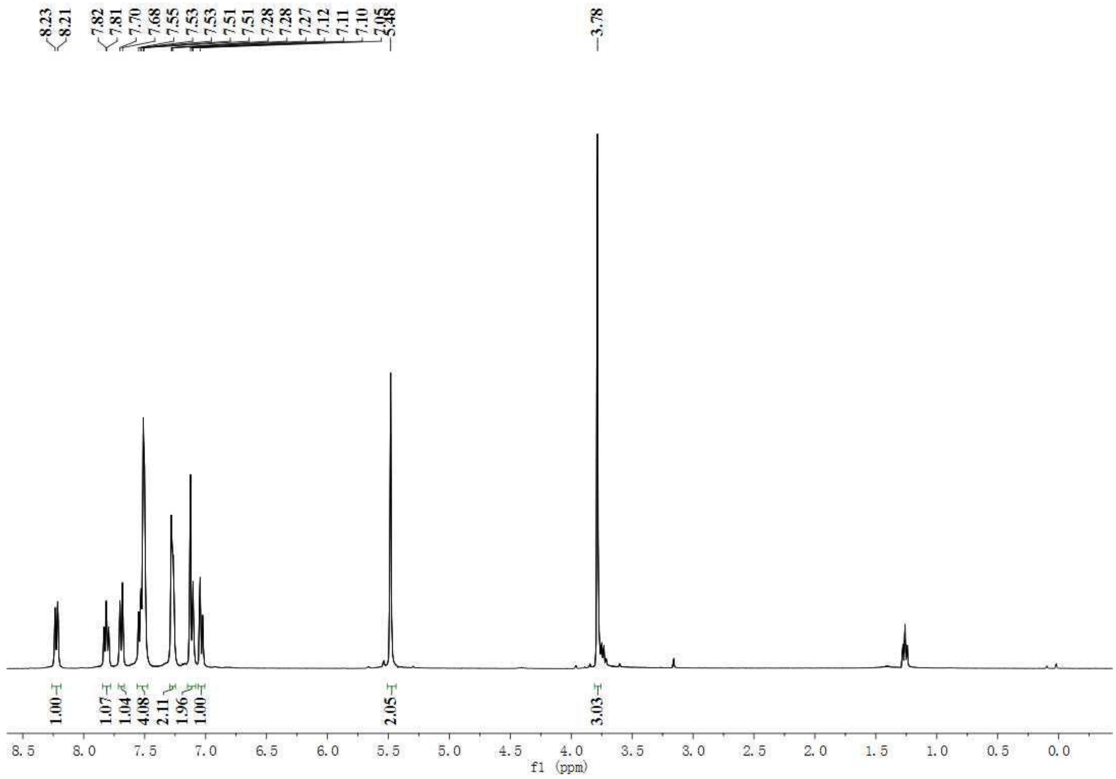

Figure S33. ^{1}H NMR spectrum of **4e**.

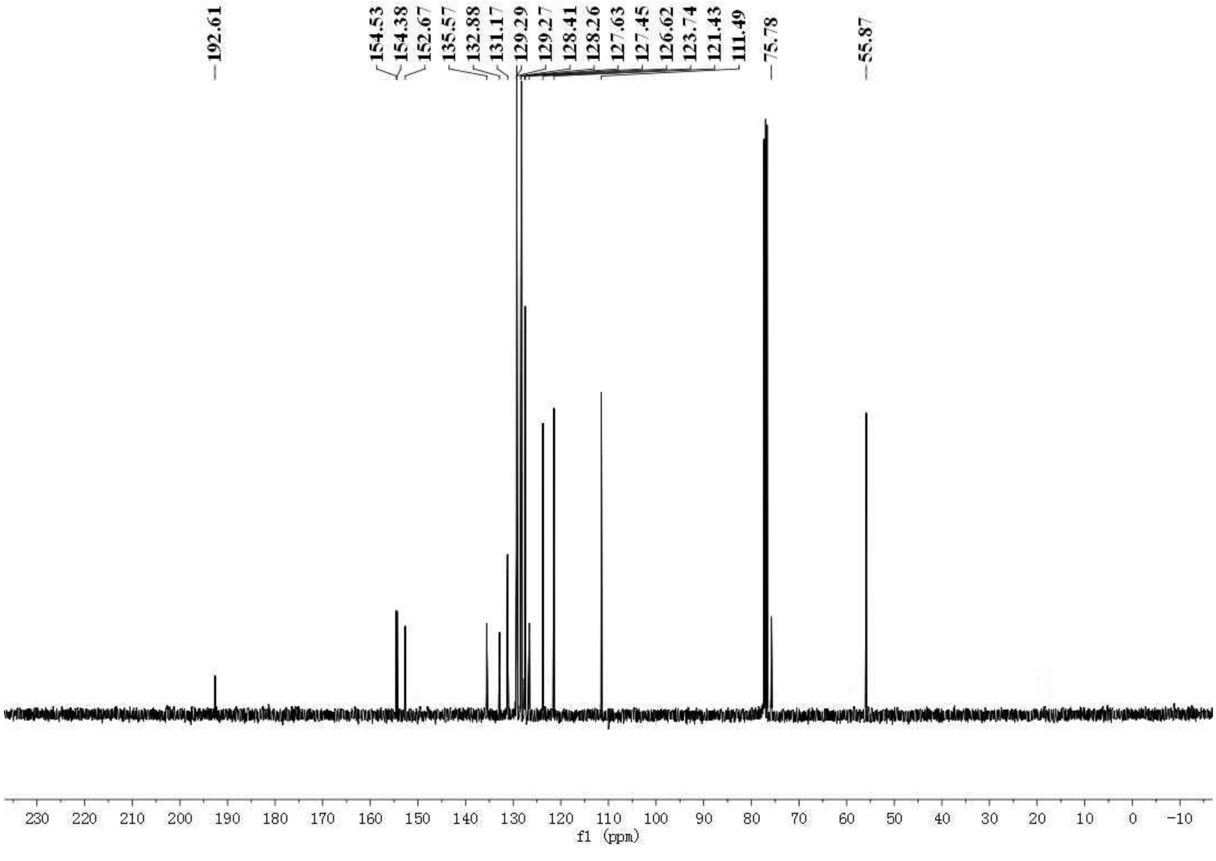

Figure S34. ^{13}C NMR spectrum of **4e**.

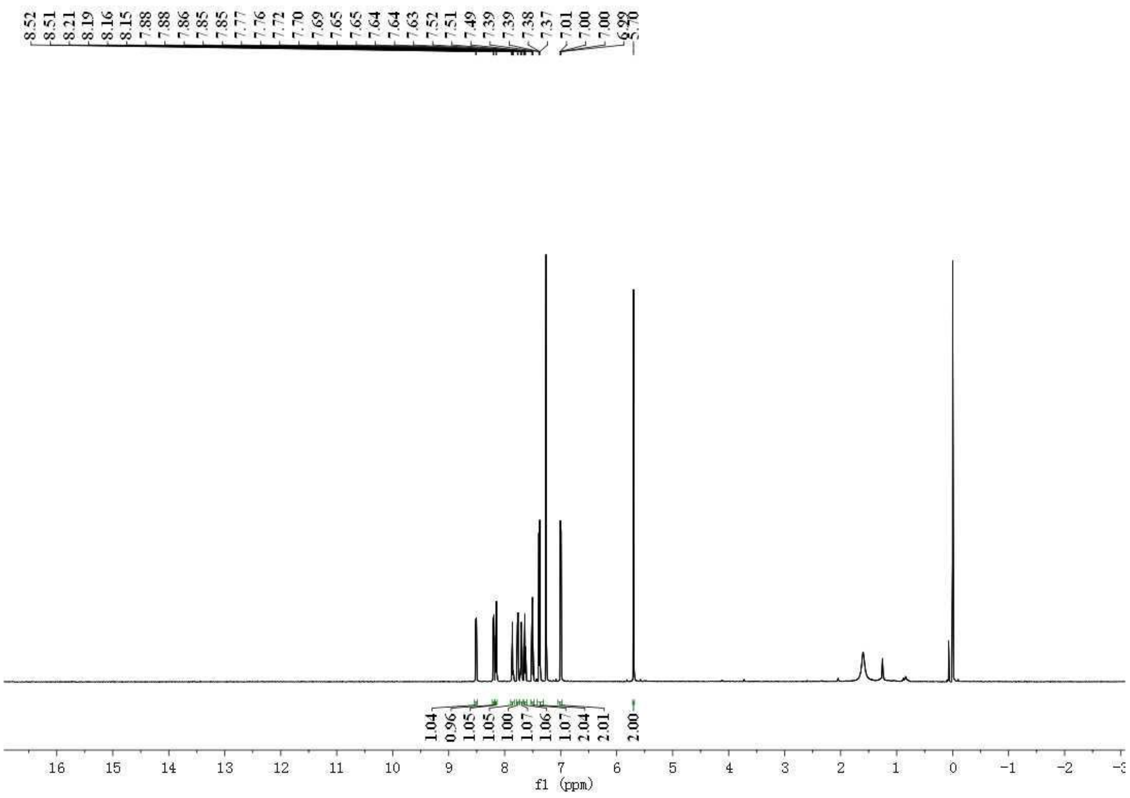

Figure S35. ^{1}H NMR spectrum of **4f**.

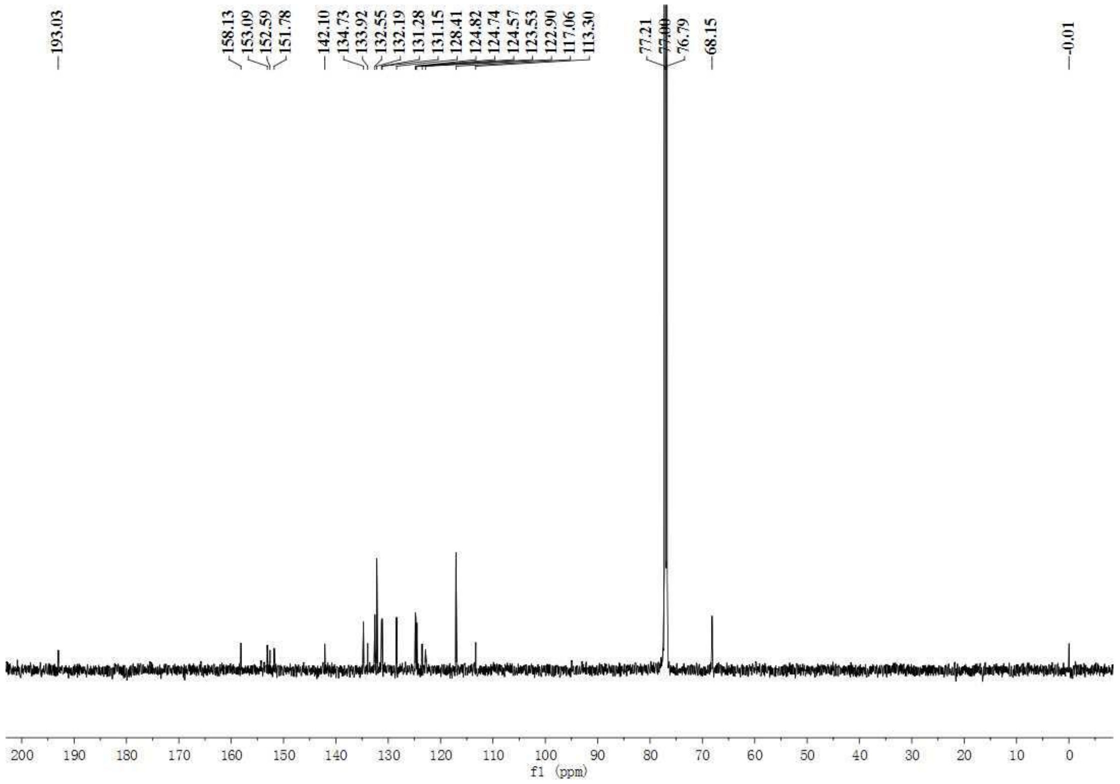

Figure S36. ^{13}C NMR spectrum of **4f**.

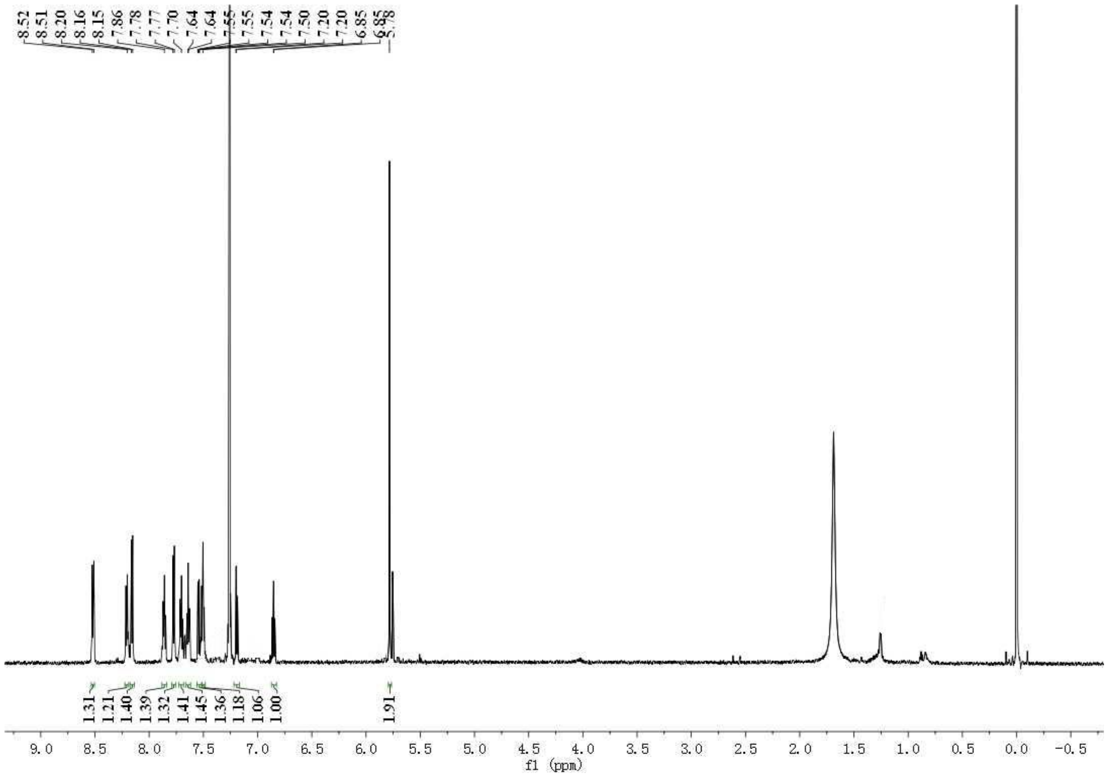

Figure S37. ^{1}H NMR spectrum of **4g**.

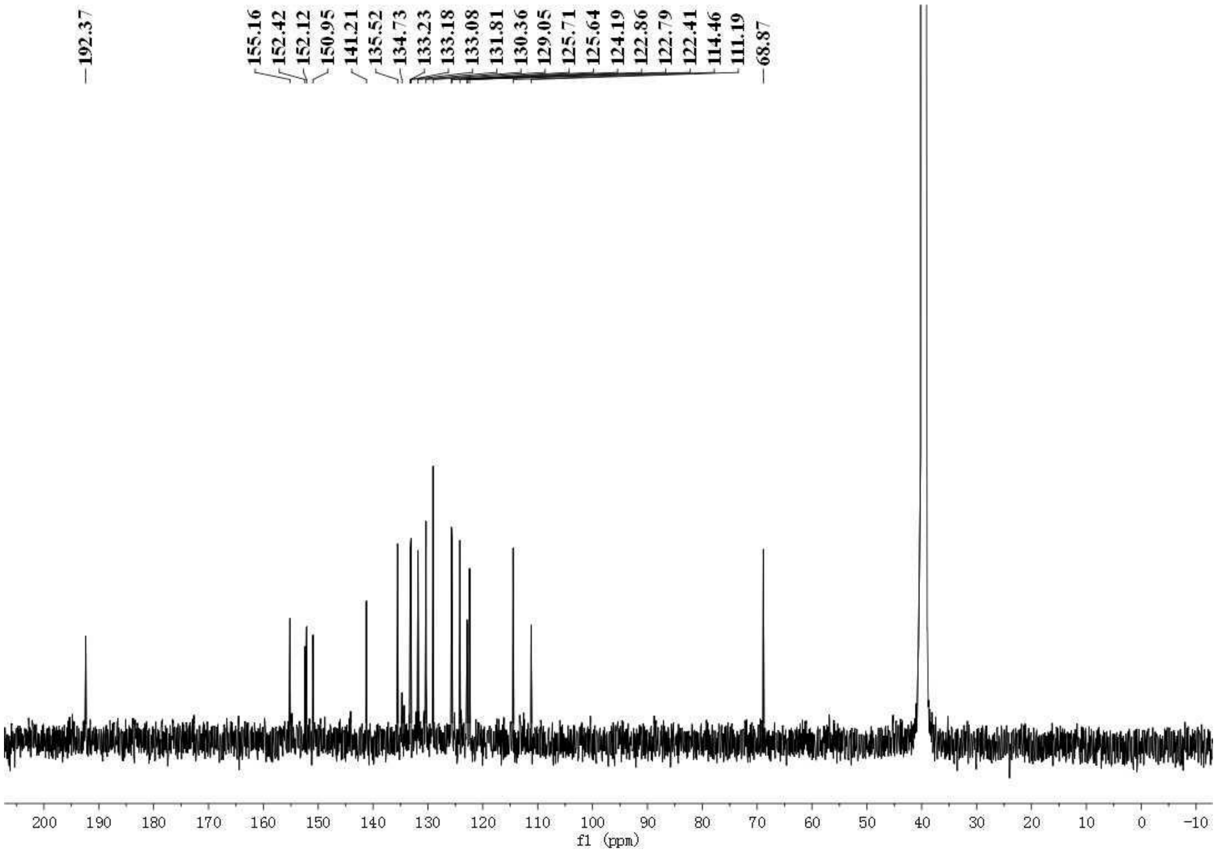

Figure S38. ^{13}C NMR spectrum of **4g**.

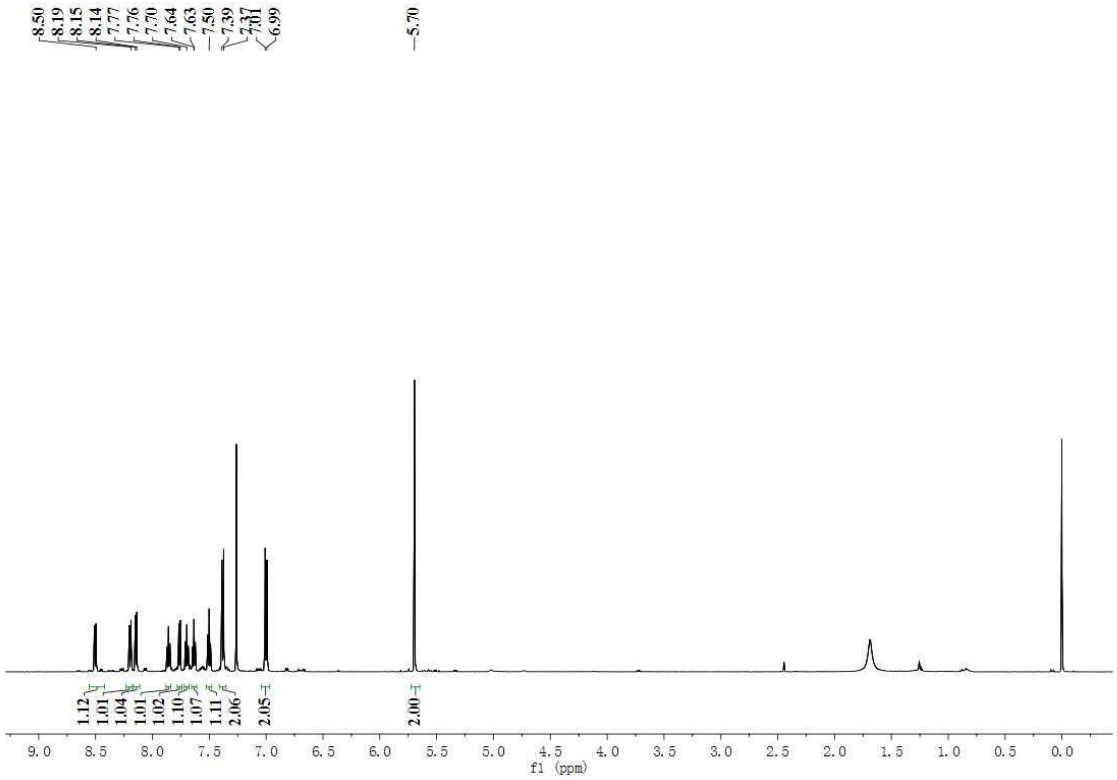

Figure S39. ^{1}H NMR spectrum of **4h**.

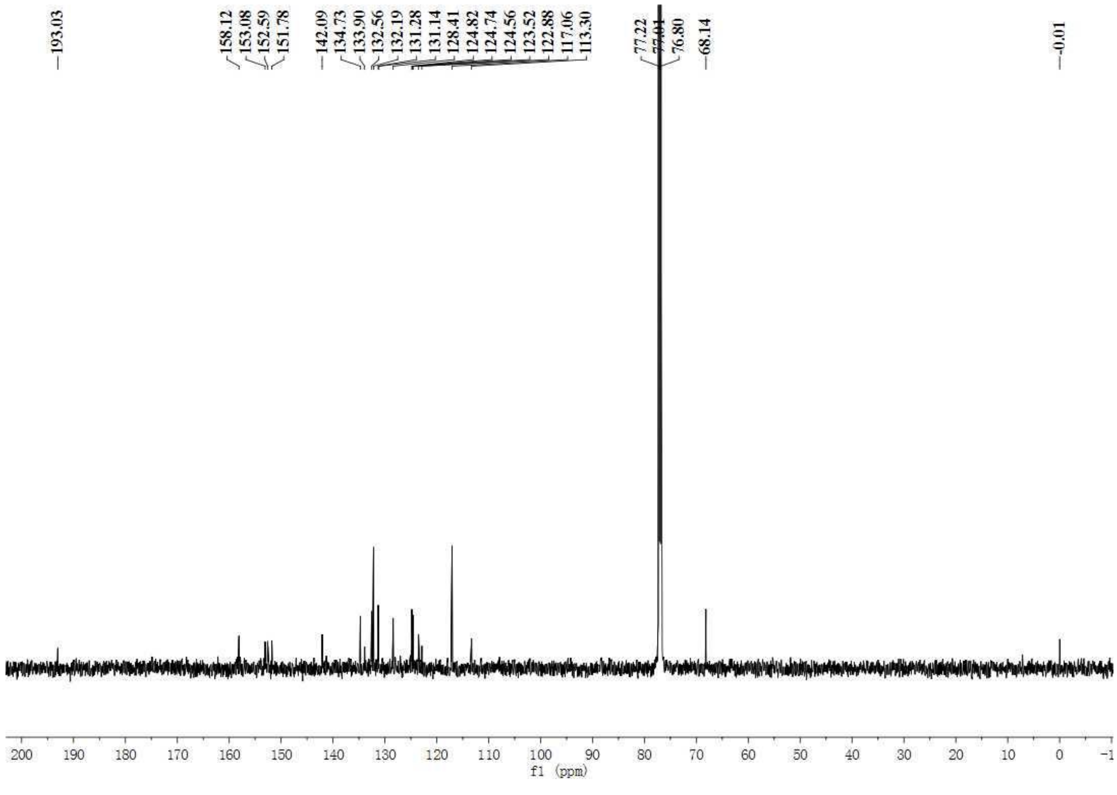

Figure S40. ^{13}C NMR spectrum of **4h**.

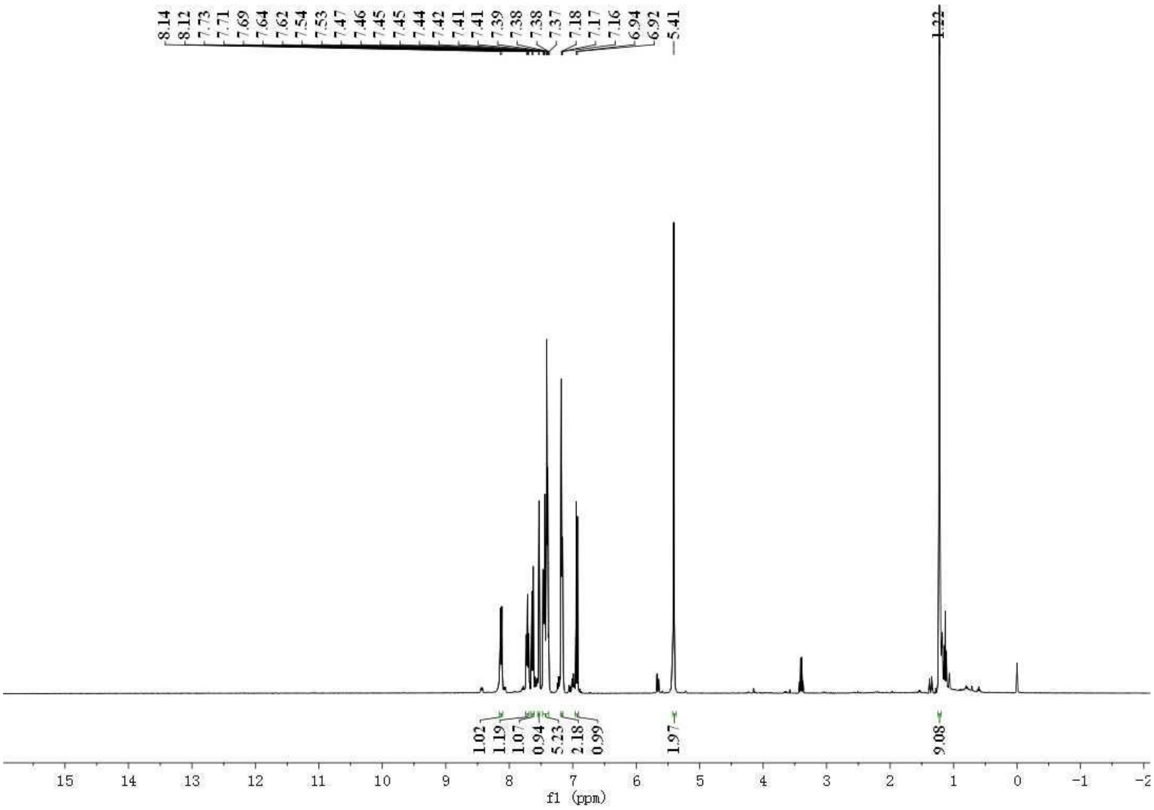

Figure S41. ^{1}H NMR spectrum of **4i**.

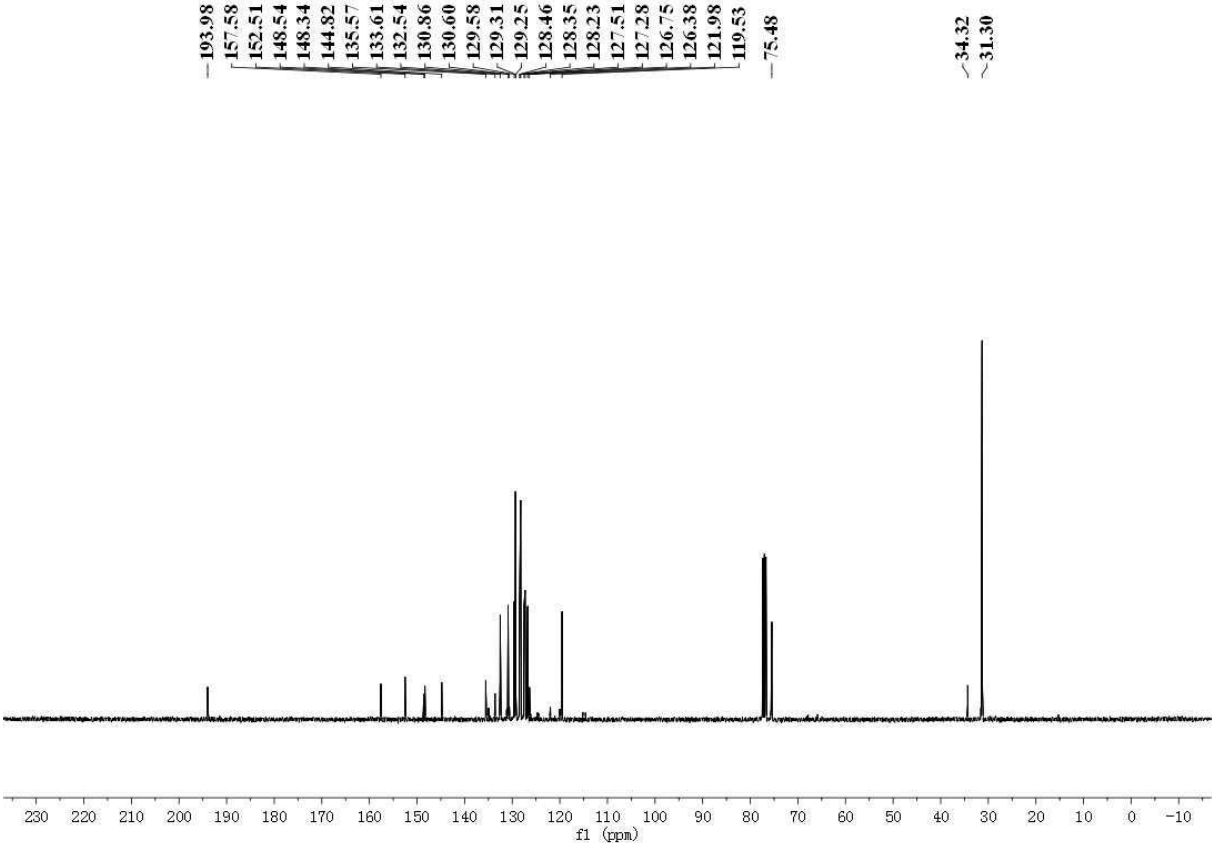

Figure S42. ^{13}C NMR spectrum of **4i**.

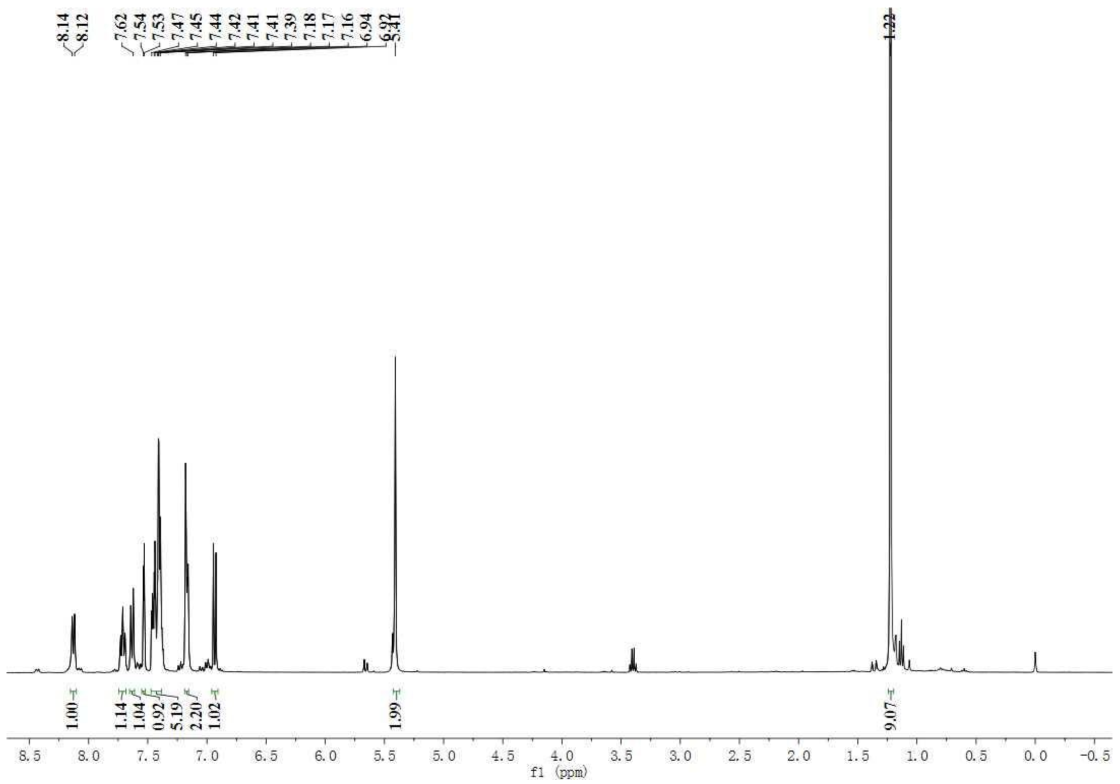

Figure S43. ^{1}H NMR spectrum of **4j**.

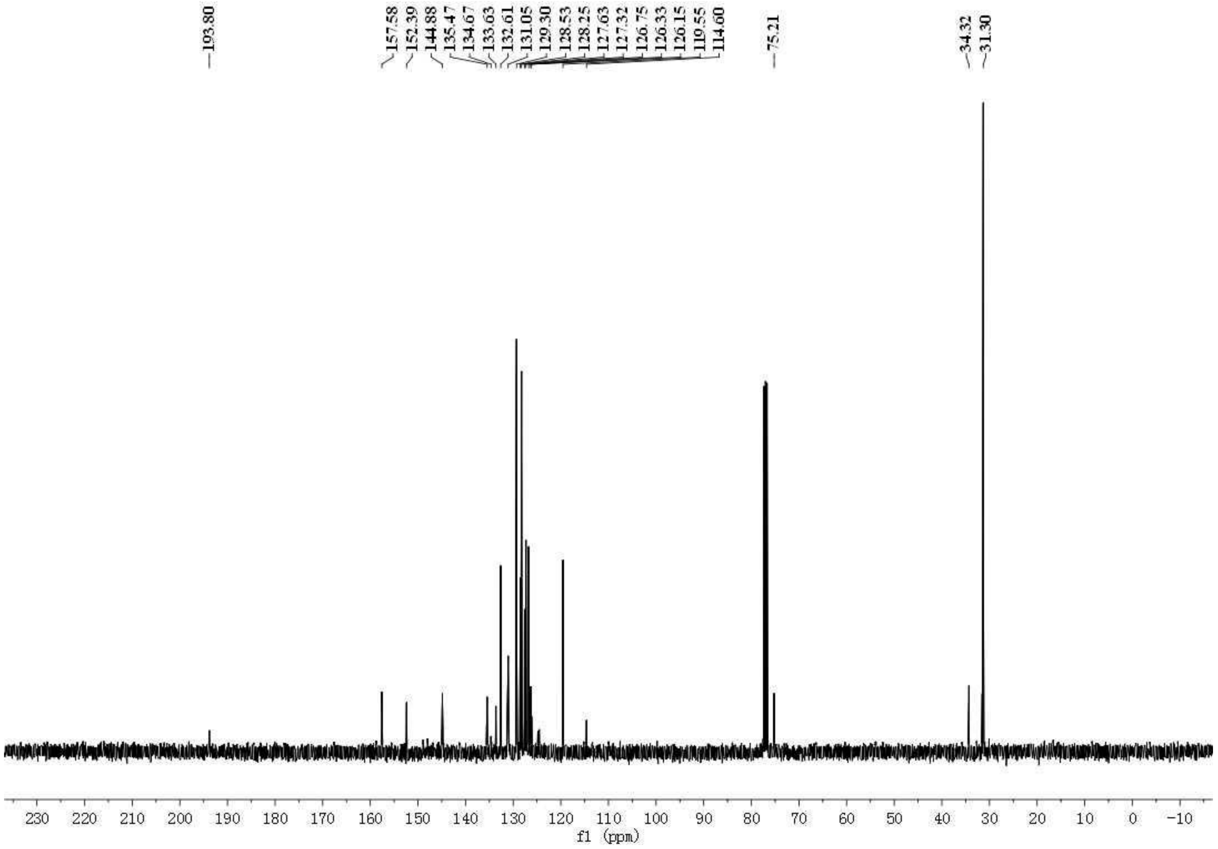

Figure S44. ^{13}C NMR spectrum of **4j**.

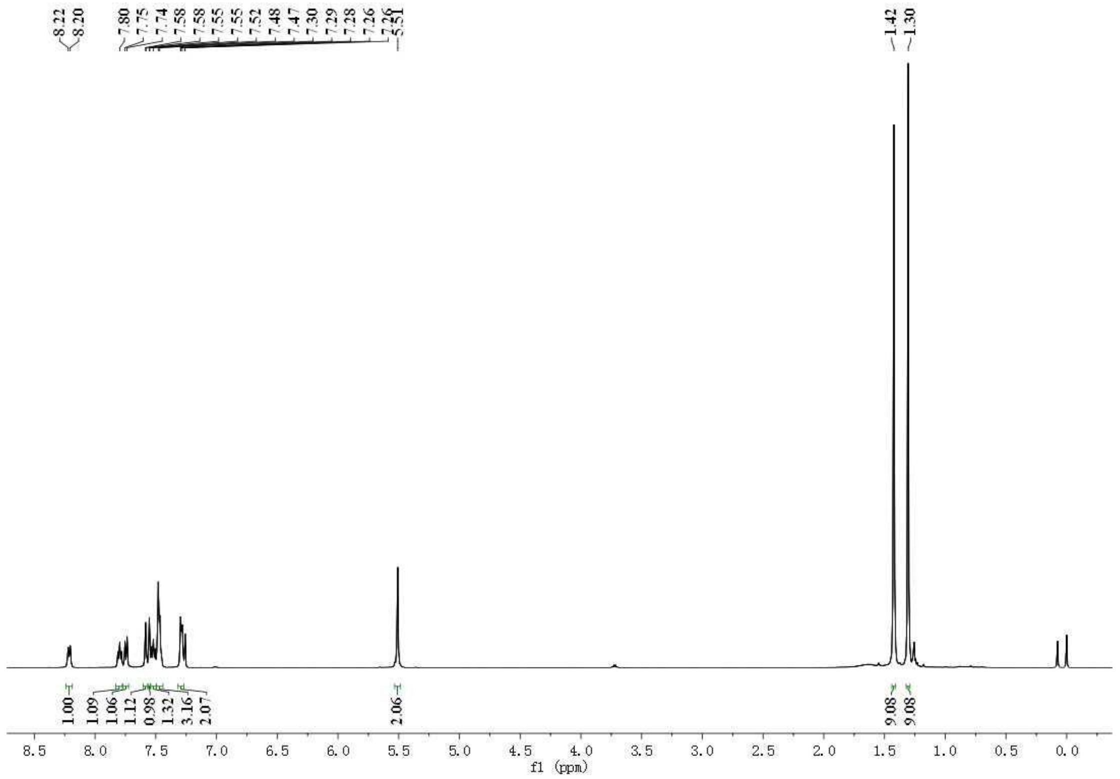

Figure S45. ^{1}H NMR spectrum of **4k**.

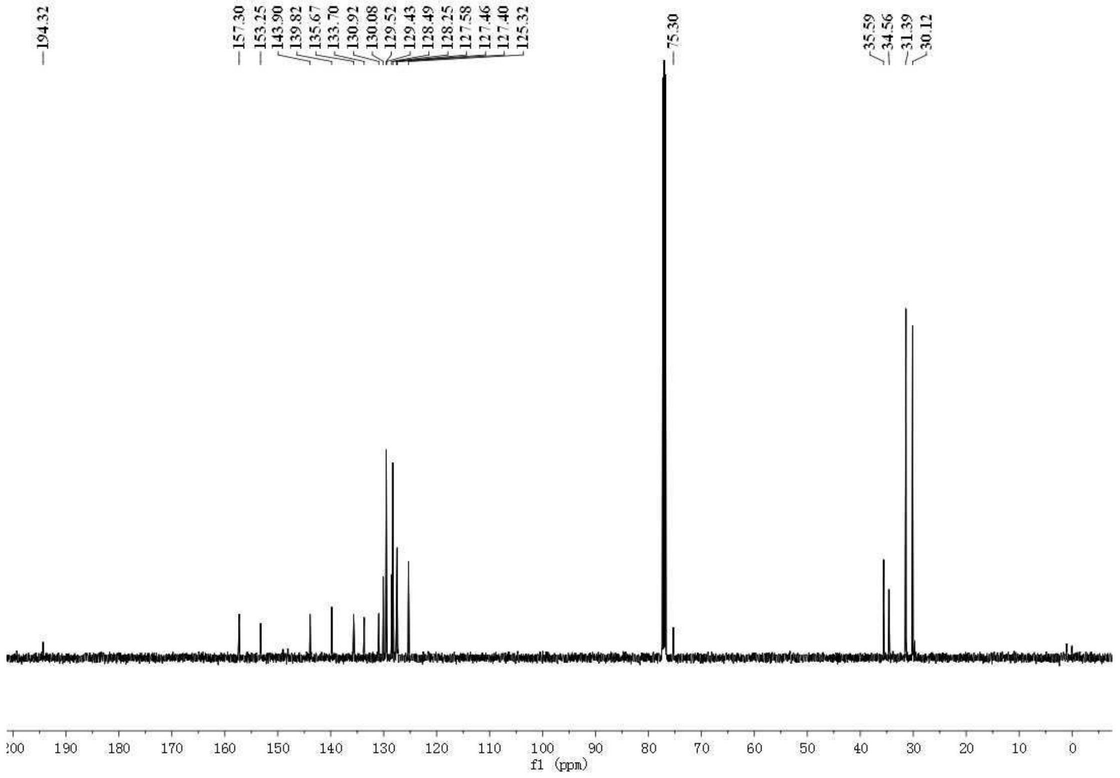

Figure S46. ^{13}C NMR spectrum of **4k**.

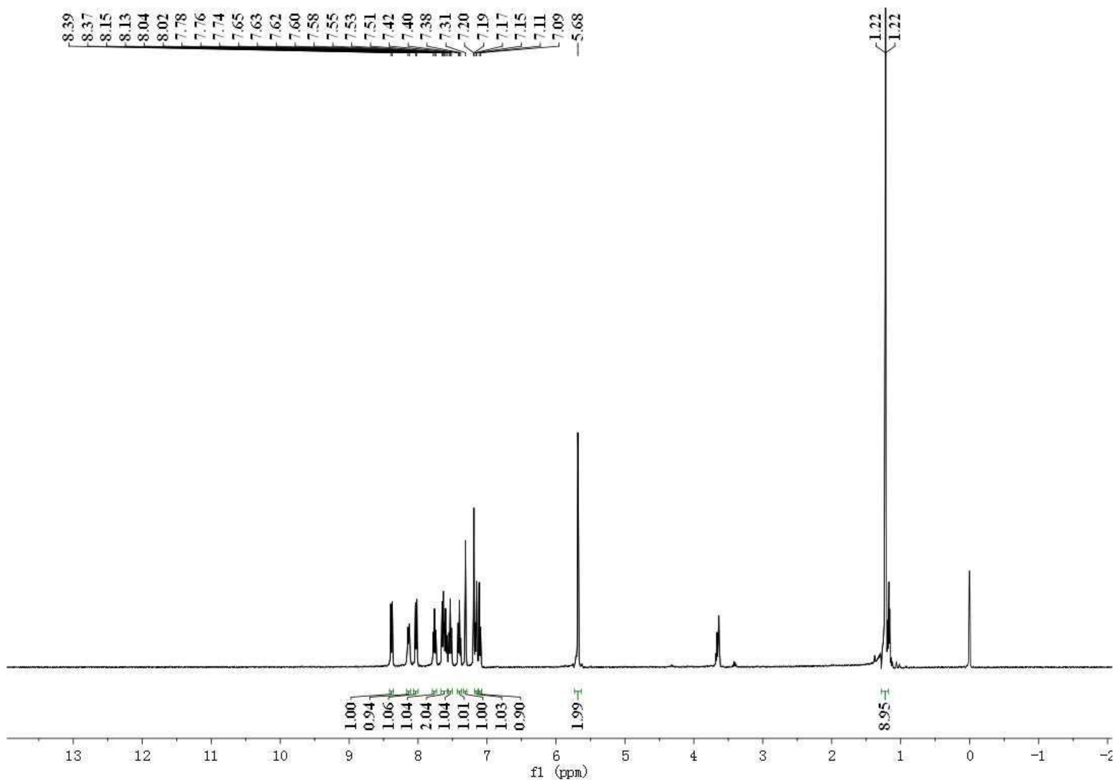

Figure S47. ^{1}H NMR spectrum of **4l**.

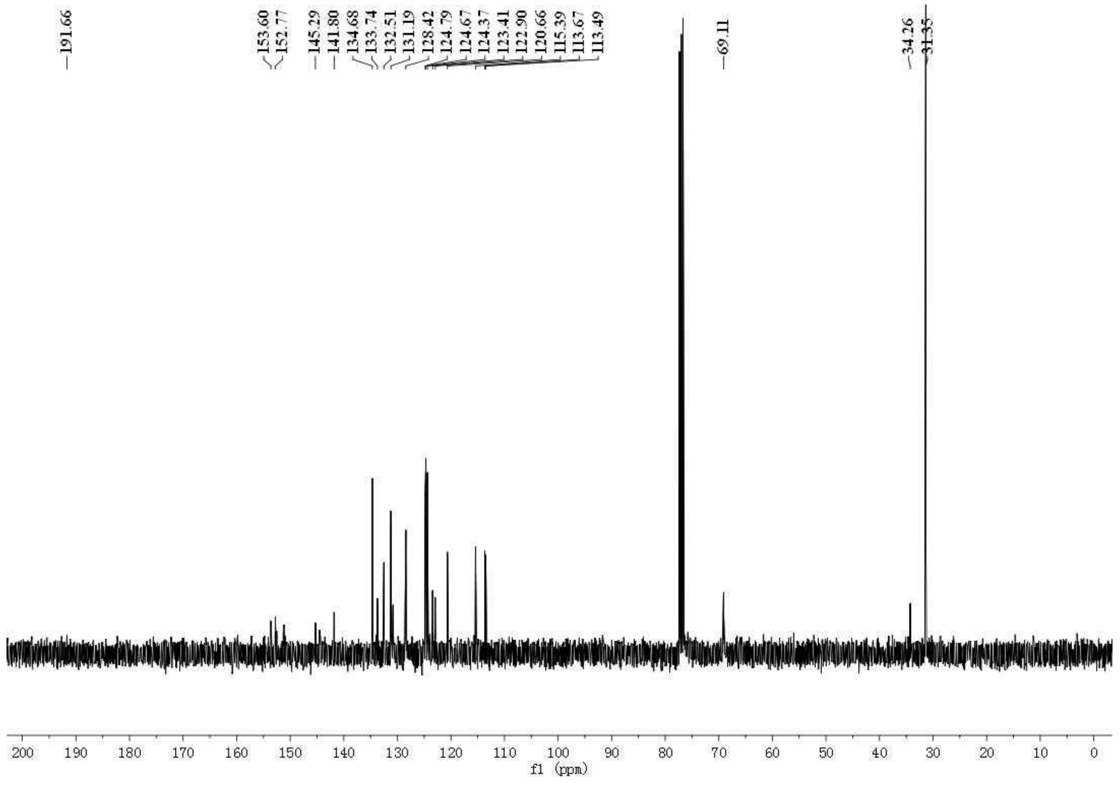

Figure S48. ^{13}C NMR spectrum of **4l**.

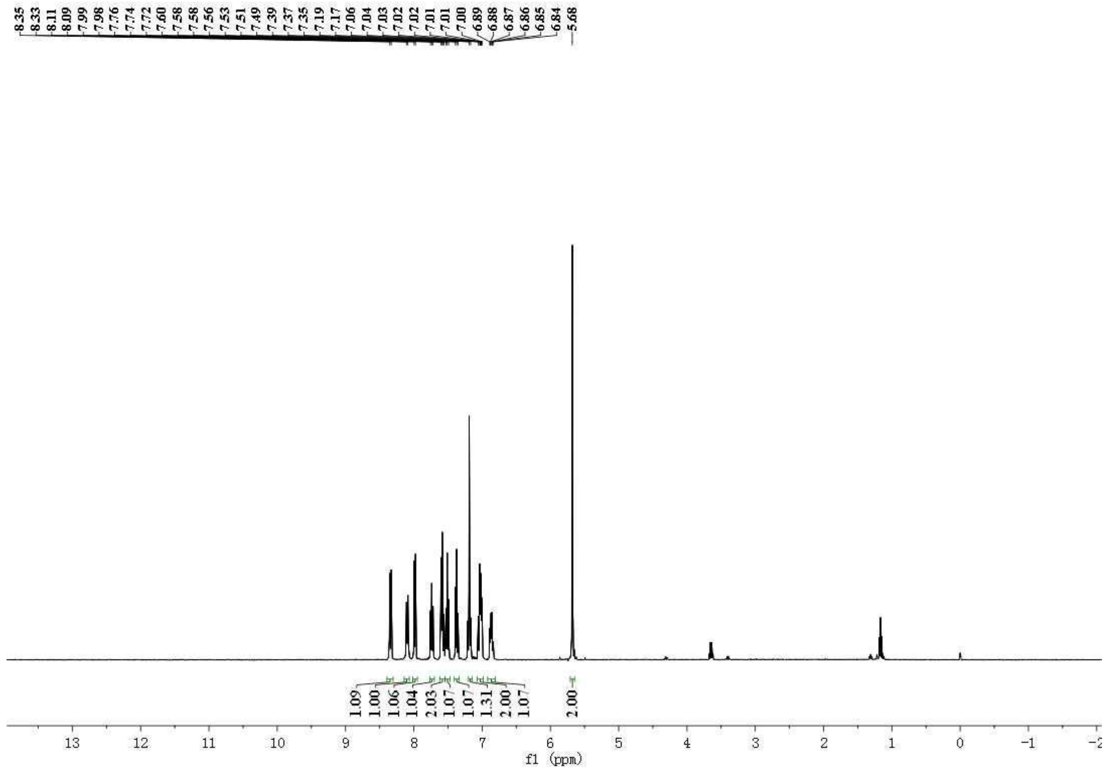

Figure S49. ^{1}H NMR spectrum of **4l′**.

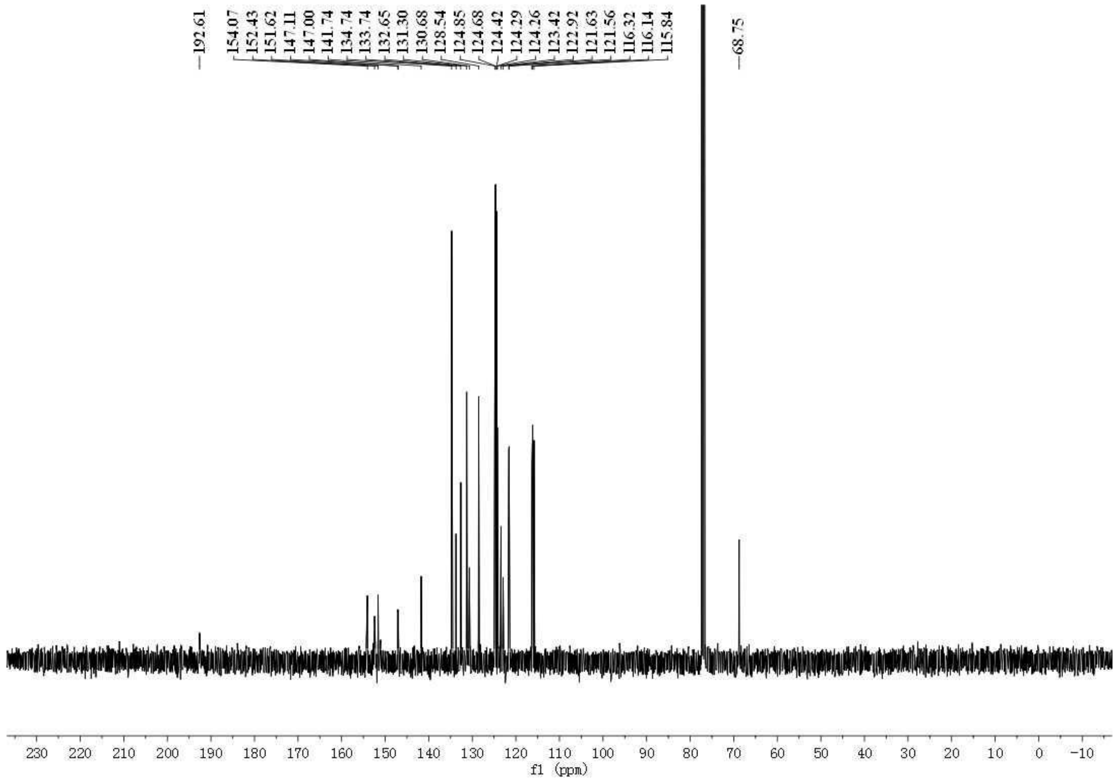

Figure S50. ^{13}C NMR spectrum of **4l′**.
